# The dual role of glioma exosomal microRNAs: glioma eliminates tumor suppressor miR-1298-5p via exosomes to promote immunosuppressive effects of MDSCs

**DOI:** 10.1038/s41419-022-04872-z

**Published:** 2022-05-02

**Authors:** Yanhua Qi, Chuandi Jin, Wei Qiu, Rongrong Zhao, Shaobo Wang, Boyan Li, Zongpu Zhang, Qindong Guo, Shouji Zhang, Zijie Gao, Shulin Zhao, Ziwen Pan, Yang Fan, Zihang chen, Huizhi Wang, Jianye Xu, Lin Deng, Shilei Ni, Jian Wang, Hao Xue, Fuzhong Xue, Gang Li

**Affiliations:** 1 Department of Neurosurgery, Qilu Hospital, Cheeloo College of Medicine and Institute of Brain and Brain-Inspired Science, Shandong University, 250012 Jinan, Shandong China; 2 Shandong Key Laboratory of Brain Function Remodeling, 250012 Jinan, Shandong China; 3 Institute for Medical Dataology of Shandong University, Jinan, People’s Republic of China; 4 Department of Biomedicine, University of Bergen, Jonas Lies vei 91, 5009 Bergen, Norway; 5 Department of Epidemiology and Health Statistics, School of Public Health, Shandong University, Jinan, Shandong Province People’s Republic of China

**Keywords:** CNS cancer, Cancer microenvironment, Tumour immunology

## Abstract

Clear evidence shows that tumors could secrete microRNAs (miRNAs) via exosomes to modulate the tumor microenvironment (TME). However, the mechanisms sorting specific miRNAs into exosomes are still unclear. In order to study the biological function and characterization of exosomal miRNAs, we performed whole-transcriptome sequencing in 59 patients’ whole-course cerebrospinal fluid (CSF) small extracellular vesicles (sEV) and matched glioma tissue samples. The results demonstrate that miRNAs could be divided into exosome-enriched miRNAs (ExomiRNAs) and intracellular-retained miRNAs (CLmiRNAs), and exosome-enriched miRNAs generally play a dual role. Among them, miR-1298-5p was enriched in CSF exosomes and suppressed glioma progression in vitro and vivo experiments. Interestingly, exosomal miR-1298-5p could promote the immunosuppressive effects of myeloid-derived suppressor cells (MDSCs) to facilitate glioma. Therefore, we found miR-1298-5p had different effects on glioma cells and MDSCs. Mechanically, downstream signaling pathway analyses showed that miR-1298-5p plays distinct roles in glioma cells and MDSCs via targeting SETD7 and MSH2, respectively. Moreover, reverse verification was performed on the intracellular-retained miRNA miR-9-5p. Thus, we confirmed that tumor-suppressive miRNAs in glioma cells could be eliminated through exosomes and target tumor-associated immune cells to induce tumor-promoting phenotypes. Glioma could get double benefit from it. These findings uncover the mechanisms that glioma selectively sorts miRNAs into exosomes and modulates tumor immunity.

## Introduction

Glioma is the most common primary CNS tumor and has a very poor prognosis, with an average median survival of ~14–17 months [1, 2]. The malignancy of tumors is not only determined by cancer cells themselves but also depends on the cross-talk between the tumors and their microenvironment [3]. TME consists of many different types of immune cells, including tumor-associated macrophages and microglia (TAMs), dendritic cells (DCs), myeloid-derived suppressor cells (MDSCs), neutrophils, and lymphoid cells [3–5]. Exosomes play an essential role in mediating intercellular communication between tumors and TME [6]. Exosomes are 30–150 nm nanovesicles, which originate from the endosome and contain nucleic acids, lipids, and proteins. Extensive evidence from recent studies showed that miRNAs in exosomes are important for tumor diagnosis and treatment [7]. Exosomal miRNAs could modulate tumor proliferation, migration, angiogenesis, and the epithelial-mesenchymal transition (EMT) as tumor suppressors or promoters. Moreover, exosomal miRNAs could even affect the TME, influencing the immune cells activation and recruitment [8].

In our previous study, we found that glioma delivered tumor-promoting miRNAs to immune cells in the TME via exosomes to induce the immune-suppressing type of immune cells, further promoting the tumor progression [9–12]. However, it is not completely clear whether tumor-suppressor miRNAs in exosomes still play a tumor-suppressive role in the TME.

It is reported that compared with the normal brain, a variety of tumor-suppressive miRNAs of glioblastoma is significantly downregulated. However, the mechanism is still not clear [13]. In the present study, we sequenced the CSF exosomes and matched tumor tissue samples from 59 patients. And we found that miRNAs highly enriched in glioma exosomes were almost tumor-suppressive miRNAs. Some tumor-suppressive miRNAs are always sorted explicitly into exosomes to downregulate their expression level, and some tumor-promoting miRNAs were almost stuck in tumor cells. We conducted research on representative tumor-suppressive EXOmiRNA miR-1298-5p and tumor-promoting CLmiRNA miR-9-5p. Results showed that EXOmiRNA miR-1298-5p could inhibit the proliferation of glioma. Glioma sorted oncosuppressor miR-1298-5p into exosomes via heterogeneous nuclear ribonucleoprotein A2B1 (hnRNPA2B1). On the contrary, CLmiRNA miR-9-5p could promote the progression of glioma and be stuck inside cells.

More importantly, we have also demonstrated for the first time that these tumor-suppressive EXOmiRNAs can be taken up by immune cells in the TME and turn into cancer promoters. In our previous study, we found exosomal miRNAs could target tumor-associated immune cells to modulate tumor immunity [9, 11, 12, 14]. Therefore, we overexpressed miR-1298-5p in the macrophage and MDSCs and found miR-1298-5p could induce immunosuppressive effects of MDSCs. Thus, we demonstrated that tumor-suppressive miRNAs in glioma cells could be eliminated through exosomes and target tumor-associated immune cells to induce tumor-promoting phenotypes.

Our research has shown that miRNAs have different effects on various cell types [15]. We hypothesized that glioma can selectively excrete tumor-suppressive miRNAs from tumor cells via exosomes and deliver them to immune cells in TME to transform them into cancer-promoting phenotypes. This mechanism that tumor cells selectively sort tumor-suppressing miRNAs into exosomes to get double benefit played an important role in tumor progression, which deserved our further attention.

## Results

### RNA expression pattern of CSF sEV in glioma patients

**Fig. 1:**
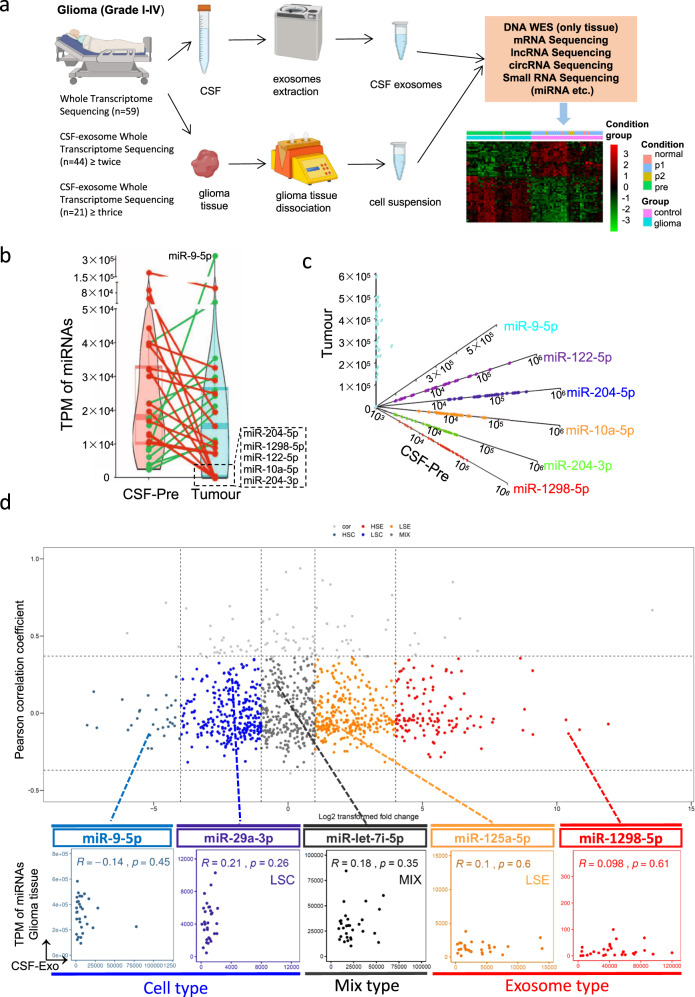
The CSF sEV miRNAs efflux. **a** Flowchart of our CSF sEV cohort study in 44 CSF sEV samples available patients with glioma from 148 WHO grade I–IV individuals. All 44 patients in this study were treated with surgery, and the tumor was totally removed under a surgical microscope. At the same time, multiple CSF specimens were obtained by a lumbar puncture at pre-operation and post-operation. Among them, the CSF specimens of 19 patients were further collected during the follow-up. These tumor specimens were undergoing whole exon and whole-transcriptome sequencing, and the CSF samples were extracting sEV for following whole-transcriptome sequencing. **b** Violin-plot showing pair-wise comparison of high-expressed miRNAs (TPM > 10000) expression in pre-operation CSF sEV and glioma tissues. At left: each point represents the mean value of high-expressed miRNAs in pre-operation CSF sEV. At right: each point represents the mean value of high-expressed miRNAs in glioma tissues. Points of same miRNAs at left and right were link together. **c** A combination of scatter diagram of miR-9-5p, miR-122-5p, miRNA-204-5p, miR-10a-5p, miR-204-3p, miR-1298-5p. **d** Fold change and pair-wise Pearson Correlation Coefficient of miRNA expression between pre-operation CSF sEV and glioma tissues were calculated. miRNAs were divided into five types: high-selective sEV type (HSE), low-selective sEV type (LSE), mix type (MIX), low-selective cell type (LSC), and high-selective cell type (HSC). For each type, a scatter diagram of representative miRNA is displayed.

We performed whole-transcriptome sequencing on CSF sEV collected preoperatively, at 1 week, and every 3 months postoperatively as well as on the surgical excision samples of 44 glioma patients (Fig. 1a). Together with 15 control, including 12 normal brain tissues and 3 normal CSF, we initially organized a whole-transcriptome sequencing glioma cohort integrated whole-course CSF sEV RNA datasets (Fig. S1a, Table S1). The expression profile of various RNAs (miRNAs, mRNAs, lncRNAs, and circRNAs) in CSF was obtained. The miRNAs were abundant in sEV and their expression was stable and had no apparent individual difference (Fig. S2a). In contrast, mRNAs, lncRNAs, and circRNAs were low-abundance components in CSF sEV whose expression has significant individual differences (Fig. S2b to d). Unlike the CSF sEV RNA expression pattern, the expression of all these four types of RNAs was abundant and stable in glioma tissues (Fig. S3). The locations of the tumors in all patients were distributed throughout the brain, especially in the convex and parenchyma of the brain. The tumor burden (valuated by SPD) and distance between the tumor and CSF circulation pathway (CSF type) did not affect the expression level of CSF sEV (Fig. S1b, Table S2).

Our finding showed the mRNAs, lncRNAs, and circRNAs in CSF sEV all showed poor ability to indicate tumor-burdened status (Fig. S4a, b, c). These long-chain RNAs had indicative abilities in only tumor tissues and were more suitable for tumor specimen biopsies, not liquid biopsies (Fig. S5). Taken together, only sEV miRNAs could serve as biomarkers for indicating tumor-burdened status in CSF liquid biopsy. In addition, the number of days from operation to the time of first post-operative CSF collection should be more than a week to make sure the clearance of preoperative glioma sEV (Fig. S4d, e).

### The miRNAs sEV “trash can”

After differential expression analysis, a total of 443 differentially expressed miRNAs (DEmiRNAs, |log2 foldchange | ≥1 and FDR < 0.05, Table S3) were identified between tumor-burdened CSF and tumor tissues, of which 313 were downregulated and 130 were upregulated (Fig. S6a). Then, mRNA targets of down- and upregulated CSF sEV DEmiRNAs in glioma were obtained (Fig. S6b) and these targets were significantly enriched for many cancer-related pathways (Fig. S6c). A total of 115 high-expressed miRNAs (transcripts per million (TPM) > 100 in post-operative CSF sEV or glioma tissues) were derived from DEmiRNAs between tumor tissue and CSF sEV. Surprisingly, the majority of these 115 miRNAs were tumor-suppressor miRNAs, although they were up or downregulated in tumor-burdened CSF sEV (Fig. S6d, Table S4).

The top 22 miRNAs (TPM > 10,000) accounted for more than 80% of the total expression, which is mostly present in the list of CSF sEV DEmiRNAs (Fig. 1b). Furthermore, some high-abundant tumor-suppressive miRNAs such as miR-1298-5p, miR-122-5p, miR-204-5p showed an absolutely excretion tendency to sEV, and some high-abundance tumor-promoting miRNAs such as miR-9-5p were almost stuck in tumor cells (Fig. 1c).

According to the log2 fold-change of miRNAs TPM values in CSF sEV and glioma tissues, we divided the sEV efflux patterns of differential expressed miRNAs into five types: high-selective sEV type (HSE, log2 foldchange > 4), low-selective sEV type (LSE, log2 foldchange > 1), mix type (MIX, 1 ≥ log2 foldchange ≥ −1), low-selective cell type (LSC, log2 foldchange < −1), and high-selective cell type (HSC, log2 foldchange < −4) (Fig. 1d).

### miR-1298-5p could inhibit the proliferation of glioma in vitro and vivo

In U87MG, U251, LN229, and P3 primary GBM cells, the expression level of imR-1298-5p was lower than normal human astrocytes (NHA) (Fig. S7a). In the TCGA and CGGA datasets, the glioma patients with higher miR-1298-5p levels had longer overall survival times (Fig. S7b, c).

**Fig. 2:**
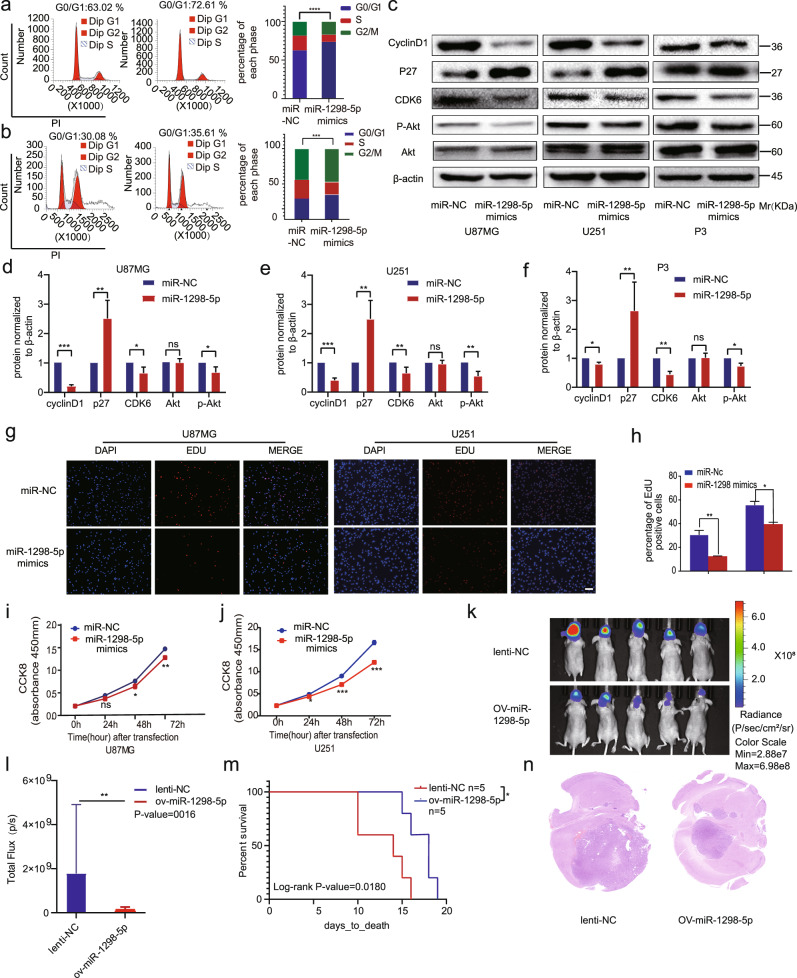
miR-1298-5p inhibited the proliferation of glioma in vitro and vivo. **a**, **b** Cell cycle analysis for U87MG and U251 cells transfected with miR-1298-5p mimics and a control sequence. The percentage of cells arrested in the G1/S phase is analyzed in a histogram (right panels). **c**–**f** The protein level of proliferation markers cyclinD1, P27, CDK6, and the AKT pathway in U87MG, U251 and P3 cells transfected with miR-1298-5p mimics and a control sequence were assessed by western blotting. Amounts of protein determined by densitometry of protein bands from three experiments.β-actin was the loading control. **g**–**j** The proliferation capacity of U87MG and U251 cells transfected with miR-1298-5p mimics were assessed using CCK8 assay and Edu assay. **k** In vivo bioluminescent imaging analysis of tumor growth in xenograft nude mice at day 10. **l** Quantification of luminescent signals in the Lenti-NC and OV-miR-1298-5p mice. **m** Survival analysis of nude mice orthotopically implanted with U87MG cells transfected with lentivirus overexpressing the control sequence or miR-1298-5p. (*P* = 0.0180 by log-rank analysis; data from 5 animals/group). **n** H&E staining of xenograft sections from miR-1298-5p-overexpressing or negative control U87MG cell tissues on the same day of execution. Data are shown as the mean ± SD of three independent experiments. Statistical significance was determined using one-way ANOVA test (*P < 0.05; **P < 0.01; ***P < 0.001).

We overexpressed miR-1298-5p in U87MG, U251, and P3 primary GBM cells and tested the overexpression efficiency with qRT-PCR. We found that the content of miR-1298-5p in tumor cells increased hundreds of times when it was directly transfected (Fig. S7e, f). This was far beyond the scavenging ability of tumor cells. Therefore, the tumor cells could not eliminate the miRNA when it was directly transfected. The flow cytometry assay showed that miR-1298-5p could Induce G1/S arrest in glioma (Fig. 2a, b). As shown in Fig. 2c–f, miR-1298-5p overexpression decreased the protein level of proliferation makers. Similarly, Edu assay and CCK8 assay were performed to examine the inhibitory effects of miR-1298-5p on cell proliferation (Fig. 2g–j).

To determine the function of miR-1298-5p in vivo, we stably overexpressed miR-1298-5p in the luciferase-labeled U87MG cells and injected them into the brains of nude mice (Fig. S7d). Bioluminescent imaging showed that miR-1298-5p-overexpressing glioma cells had lower signals (Fig. 2k, l). Additionally, the nude mice implanted with miR-1298-5p-overexpressing glioma cells had a longer survival time (Fig. 2m). Moreover, HE and IHC staining proved that miR-1298-5p resulted in a remarkable reduction in tumor growth (Figs. 2n, S7g). We overexpressed the miR-1298-5p in the U87MG, U251 and P3 cells and found that the protein level of phosphorylated Akt decreased (Fig. 2c).

### miR-1298-5p promoted the immunosuppressive effects of MDSCs

**Fig. 3:**
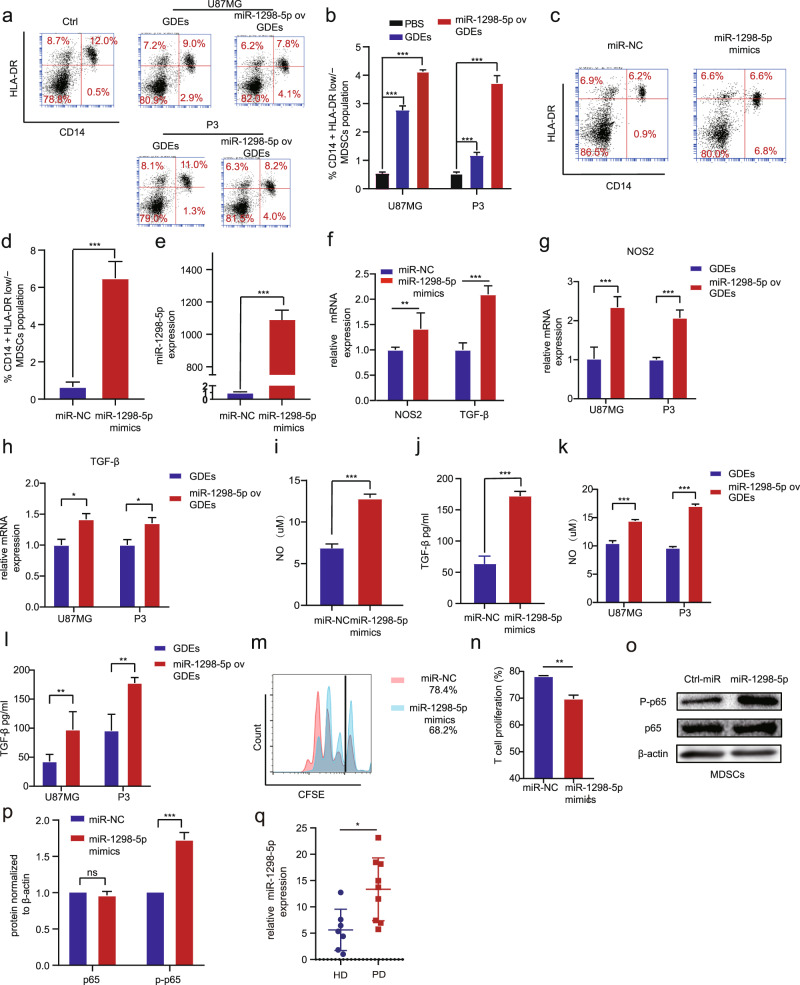
miR-1298-5p promoted the immunosuppressive effects of MDSCs. **a**, **b** Flow cytometry assay showed that GDEs overexpressing miR-1298-5p displayed a stronger MDSC induction ability than the PBS and GDEs. **c**, **d** Flow cytometry assay showed that miR-1298-5p upregulated the proportion of CD14+/HLA-DR low/-MDSCs population. **e**, **f** qRT-PCR demonstrated that miR-1298-5p increased the expression of NOS2 and TGF-β in MDSCs. **g**, **h** qRT-PCR demonstrated that GDEs overexpressing miR-1298-5p increased the expression of NOS2 and TGF-β in MDSCs. **i**, **j** NO and TGF-β in the supernatants of MDSCs were measured. **k**, **l** GDEs overexpressing miR-1298-5p increased the content of NO and TGF-β in the supernatants of MDSCs. **m**, **n** CD8 + T cell proliferation was determined by flow cytometry three days later with CFSE dilution. **o**, **p** The protein level of the NF-κB pathway in MDSCs transfected with miR-1298-5p mimics and a control sequence were assessed by western blotting. Amounts of protein determined by densitometry of protein bands from three experiments. β-actin was the loading control. **q** The expression of miR-1298-5p in MDSCs of patents was higher than health donors. Data are shown as the mean ± SD of three independent experiments. Statistical significance was determined using one-way ANOVA test (*P < 0.05; **P < 0.01; ***P < 0.001).

Next, we tried to explore whether tumor-suppressive miR-1298-5p has effects on the microenvironment. We overexpressed miR-1298-5p mimics in MDSCs. Interestingly, we found miR-1298-5p promoted the function of MDSCs (Fig. 3c, d). We isolated GDEs from the culture medium of glioma cells transfected with miR-1298-5p-overexpressing lentivirus. Transmission electron microscopy (TEM) showed that the exosomes isolated from CSF and culture supernatants of the glioma cell lines U87MG and P3 were rounded particles ranging from 30 to 100 nm (Fig. S8a). Western blot analysis revealed the presence of TSG101, CD9 and the absence of calnexin in GDEs (Fig. S8b). Then, qNano analysis determined exosome concentration and size distribution (Fig. S8c). Confocal microscopy showed the internalization of PKH67-labeled glioma exosomes (green) by MDSCs (Fig. S8d). And the GDEs overexpressing miR-1298-5p displayed a stronger MDSC induction ability than the PBS and GDEs (Fig. 3a, b). We also measured pri-miR-1298-5p in MDSCs upon exosome treatment and excluded endogenous transcription to support direct delivery (Fig. S8n). Compared to MDSCs, the content of pri-miR-1298-5p in exosomes was very low (Fig. S8o). Therefore, pri-miRNA is not increased in MDSC upon treatment with exosomes. Moreover, the expression of NOS2 and TGF-β mRNA was increased after being transfected with miR-1298-5p mimics (Fig. 3e, f). We next measured the NO and TGF-β production in MDSCs and found that NO and TGF-β were upregulated in MDSCs supernatants (Fig. 3i, j). Similarly, the GDEs overexpressing miR-1298-5p also increased the expression of NO and TGF-βin MDSCs. (Fig. 3g, h, k, l) We cultured MDSCs with T cells to evaluate the T cell-suppressing activity and found that MDSCs transfected with miR-1298-5p were more effective at suppressing T cells than the control group (Fig. 3m, n). We performed the GO and KEGG enrichment analyses and found that miR-1298-5p could regulate the NF-κB pathway in MDSCs (Fig. S13). miR-1298-5p upregulated the expression of p-p65 in MDSCs with western blot assay (Fig. 3o, p). We detected the expression of miR-1298-5p in MDSCs of patients and found that compared with healthy donors, miR-1298-5p had higher expression in the patients’ samples (Fig. 3q). Moreover, we treated the MDSCs with exosomes isolated from CSF and found that they could also promote the immunosuppressive effects of MDSCs (Fig. S9a, b). Consistent with our previous results, exosomes isolated from CSF increased the expression of NO and TGF-βin MDSCs (Fig. S9c–f).

Moreover, we treated tumor cells with miR-1298-5p-enriched exosomes and performed the qRT-PCR for miR-1298-5p. And we found that miR-1298-5p-enriched exosomes didn’t increase the expression of miR-1298-5p in tumor cells. However, when we treated tumor cells with miR-1298-5p-enriched exosomes and GW4869 (inhibitor of exosome biogenesis/release), the expression of miR-1298-5p in tumor cells was significantly increased (Fig. S14f). We also proved it with Cy3-miR-1298-5p experiment (Fig. S14e). We performed the Edu and CCK8 assays and found that miR-1298-5p-enriched exosomes did not affect the behavior of tumor cells (Fig. S14a–d). These results showed that tumor cells can escape the effects of such exosomal miRNAs in the CSF by eliminating them via exosomes again.

Collectively, these findings suggested that miR-1298-5p promoted the immunosuppressive effects of MDSCs via the NF-κB pathway.

### miR-1298-5p was sorted into exosomes via hnRNPA2B1

**Fig. 4:**
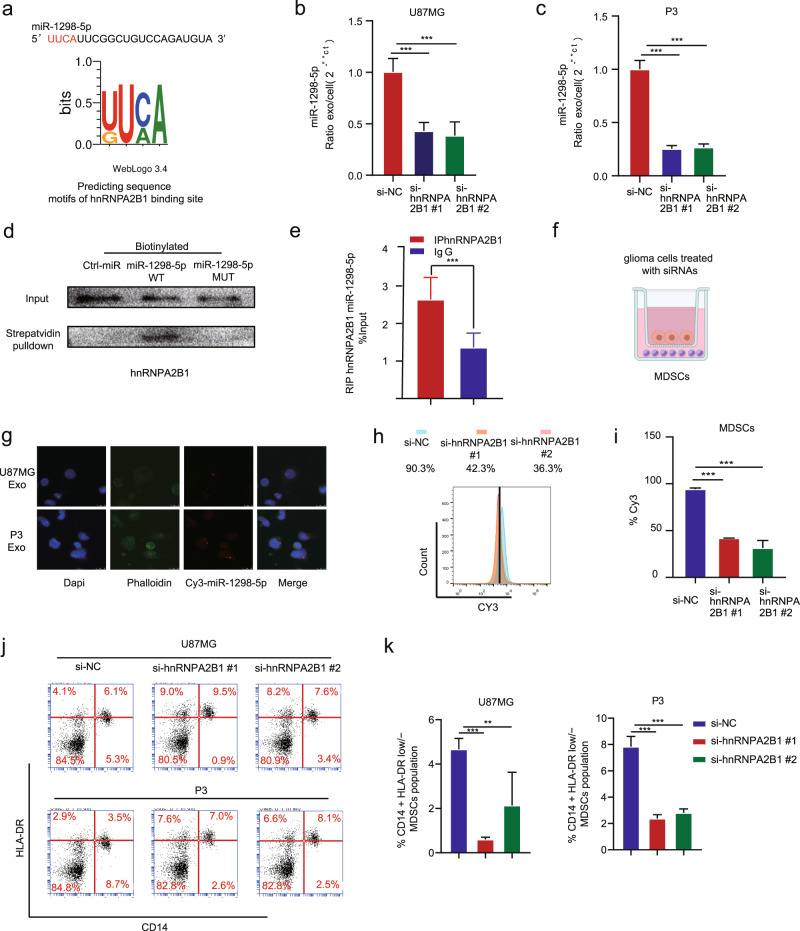
miR-1298-5p was sorted into exosomes via hnRNPA2B1. **a** Sequence motifs of hnRNPA2B1 binding site predicted by POSTAR2. **b**, **c** qRT-PCR assay analyzed the exo/cell ratio of miR-1298-5p in U87MG and P3 after knocking down hnRNPA2B1. **d** RNA pull-down and western blot with U87MG lysate confirmed that miR-1298-5p was associated with hnRNPA2B1. **e** RIP analysis using the anti-hnRNPA2B1 antibody revealed that miR-1298-5p interacted with hnRNPA2B1 in U87MG cells. The negative control, IgG. **f** Schematic graph of in vitro coculture system. **g** Internalization of Cy3-labeled miR-1298-5p by MDSCs. **h**, **i** hnRNPA2B1 was knocked down in U87MG. Internalization of Cy3-labeled miR-1298-5p by MDSCs was assessed by flow cytometry after coculture for 24 h. **j**, **k** Cultured MDSCs with U87MG and P3 knocking down hnRNPA2B1 and analyzed the ratio of CD14 + HLA-DR low/− MDSCs population using Flow cytometry assay. Statistical significance was determined using one-way ANOVA test (*P < 0.05; **P < 0.01; ***P < 0.001).

hnRNPA2B1 has been reported to sort RNAs into exosomes as RBPs selectively [16]. Sequence analysis by POSTAR2 indicated a sequence motif and structural preference of the RBP binding site for hnRNPA2B1, which was located in the miR-1298-5p (Fig. 4a) [17]. Next, we knocked down hnRNPA2B1 in U87MG and P3. And we found that hnRNPA2B1 knockdown reduced the exo/cell ratio of miR-1298-5p (Fig. 4b, c). Moreover, we knocked down hnRNPA2B1 in U87MG and P3 and measured pri-miR-1298-5p in tumor cells and exosomes. These results demonstrated that miR-1298-5p was actually transcribed but then eliminated through exosomes (Fig. S8g–j). Compared to the cells, the content of pri-miRNA in exosomes was very low (Fig. S8m). The structure of pri-miR-1298-5p is different from miR-1298-5p and the hnRNPA2B1 couldn’t bind it. We proved it with the RIP assay (Fig. S8k, l). This explained why there is no change in their abundance upon hnRNPA2B1 silencing. We tested the efficiency of hnRNPA2B1 knockdown (Fig. S8e, f). hnRNPA2B1 sorts RNAs into exosomes by recognizing a specific motif (i.e., UUCA). Therefore, we induced mutation at this site of miR-1298-5p. And then, we performed the RNA pull-down assay with biotinylated miR-1298-5p WT and MUT. We found that miR-1298-5p (WT) can bind to hnRNPA2B1 but MUT cannot (Fig. 4d). Consistently, RNA immunoprecipitation (RIP) showed enrichment of miR-1298-5p by hnRNPA2B1, validating the interaction between miR-1298-5p and hnRNPA2B1 (Fig. 4e). We transfected glioma cells with Cy3-miR-1298-5p and cocultured them with MDSCs. Finally, Cy3-miR-1298-5p was detected in MDSCs, which demonstrated that glioma cells could transfer miR-1298-5p to MDSCs via exosomes (Fig. 4g). Then, we transfected U87MG with Cy3-miR-1298-5p and knocked down hnRNPA2B1 in U87MG and cocultured them with MDSCs. The flow cytometry showed that Internalization of Cy3-labeled miR-1298-5p by MDSCs reduced (Fig. 4h, i). In addition, we knocked down hnRNPA2B1 in U87MG and P3 and cultured them with MDSCs. The results showed that hnRNPA2B1 knockdown reduced the percentage of CD14+/HLA-DR low/− MDSCs population (Fig. 4j, k).

Moreover, we performed the Edu and CCK8 assays about the phenotypic effects of hnRNPA2B1 silencing on tumor cells (Fig. S15a–d). We also performed qRT-PCR for miR-1298-5p in U87MG and U251 cells transfected with si-hnRNPA2B1 and miR-1298-5p inhibitor (Fig. S15e, f). The results showed that the effects of hnRNPA2B1 knockdown was consistent with retention of tumor-suppressive miRNAs and could be partly attenuated by miR-1298-5p inhibitors.

### miR-1298-5p targeted setd7 in glioma and MSH2 in MDSCs

**Fig. 5:**
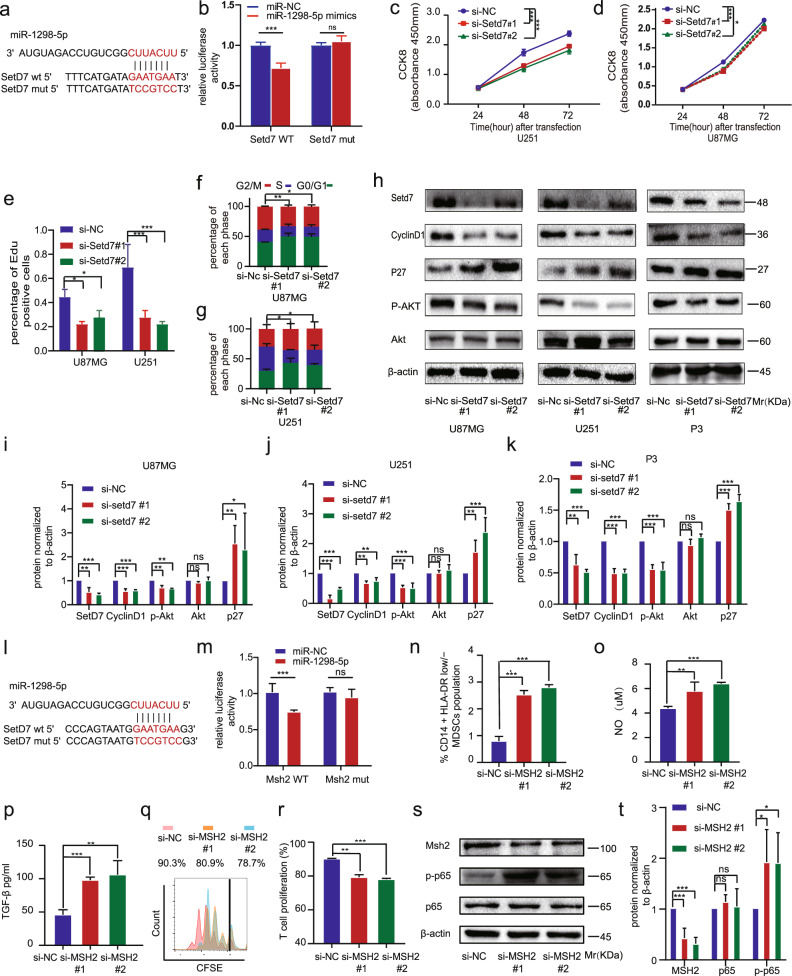
miR-1298-5p targeted SETD7 in glioma and MSH2 in MDSCs. **a** Construction of wild type (WT) and mutant type (MUT) luciferase reporter vectors based on the predicted binding site of miR-1298-5p in SETD7. **b** 293 T cells were co-transfected with the reporter vectors and miR-1298-5p or miR-Nc. Luciferase activity was assessed 48 h after transfection. **c**, **d** The proliferation capacity of U87MG and U251 cells after SETD7 knockdown were assessed using CCK8 assay. **e** The proliferation capacity of U87MG and U251 cells after SETD7 knockdown were assessed using the Edu assay. **f**, **g** Cell cycle analysis for U87MG and U251 cells knocking down SETD7. The percentage of cells arrested in the G1/S phase is analyzed in a histogram. **h**–**k** The protein level of SETD7, cyclinD1, P27, p-Akt, Akt in U87MG, U251, and P3 cells knocking down SETD7 were assessed by western blotting. Amounts of protein determined by densitometry of protein bands from three experiments. β-actin was the loading control. **l** Construction of wild type (WT) and mutant type (MUT) luciferase reporter vectors based on the predicted binding site of miR-1298-5p in MSH2. **m** 293 T cells were co-transfected with the reporter vectors and miR-1298-5p or miR-Nc. Luciferase activity was assessed 48 h after transfection. **n** Flow cytometry assay showed that MSH2 knockdown upregulated the proportion of CD14 + HLA-DR low/− MDSCs population. **o**, **p** NO and TGF-β in the supernatants of MDSCs were measured after MSH2 knockdown. **q**, **r** CD8 + T cell proliferation was determined by flow cytometry 3 days later with CFSE dilution. **s**, **t** The protein level of p65 and p-p65 in MDSCs transfected with MSH2 siRNAs were accessed by western blotting. Amounts of protein determined by densitometry of protein bands from three experiments. β-actin was the loading control. Data are shown as the mean ± SD of three independent experiments. Statistical significance was determined using one-way ANOVA test (*P < 0.05; **P < 0.01; ***P < 0.001).

We predicted the target genes of miR-1298-5p by Starbase and miRDB (Fig. S7h) [18, 19]. Next, we studied the interaction between the target genes and glioma proliferation, and setd7 was found. After transfection of miR-1298-5p, SETD7 expression was downregulated (Fig. S7i, j). We constructed the dual-luciferase reporter plasmids, including the WT and MUT 3’UTR of SETD7 (Fig. 5a). And compared with control miRNA, miR-1298-5p decreased the luciferase activity of the WT plasmid and did not affect the MUT plasmid (Fig. 5b).

**Fig. 6:**
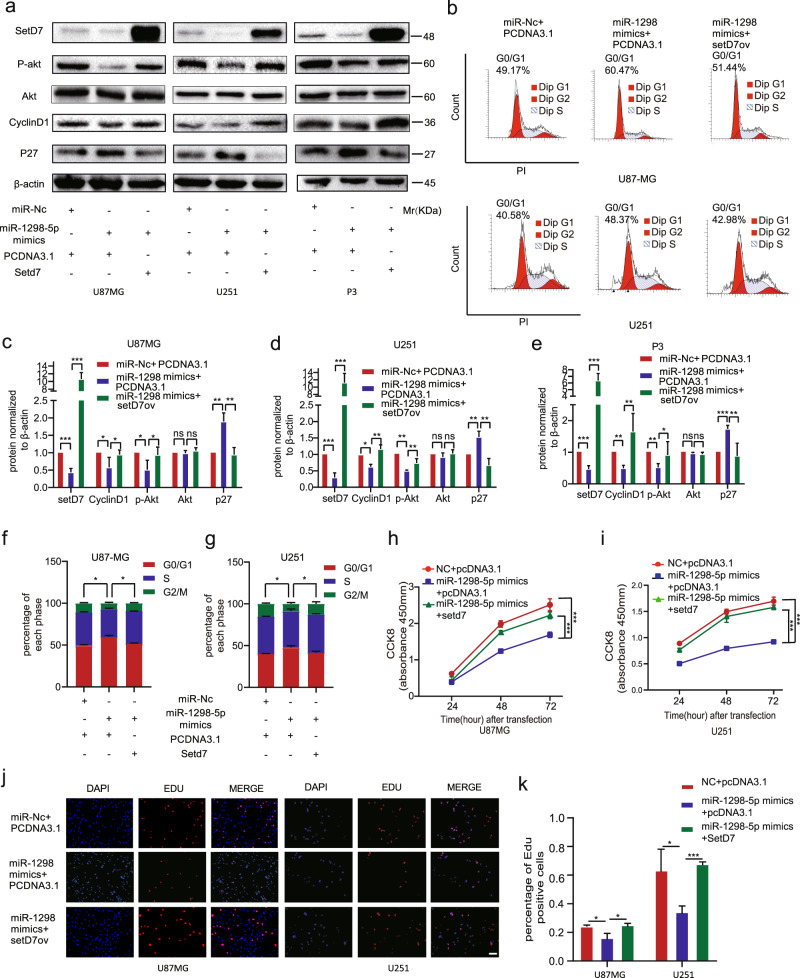
The effects of miR-1298-5p overexpression in glioma could be partially attenuated by SETD7 overexpression. **a** Protein level of SETD7, cyclinD1, P27, p-Akt, Akt in U87MG, U251, and P3 cells transfected with miR-1298-5p mimics and pcDNA3.1-SETD7 or pcDNA3.1 were assessed by western blotting. β-actin was used as the control for normalization. **b** Cell cycle analysis for U87MG and U251 cells. **c–e** Amounts of protein determined by densitometry of protein bands from three experiments. β-actin was the loading control. **f**, **g** The percentage of cells transfected with miR-1298-5p mimics arrested in the G1/S phase can be attenuated by SETD7 overexpression. **h**–**k** The proliferation capacity of U87MG and U251 cells treated as described above were assessed using CCK8 assay and EDU assay. Data are shown as the mean ± SD of three independent experiments. Statistical significance was determined using one-way ANOVA test (*P < 0.05; **P < 0.01; ***P < 0.001).

To determine the functional role of SETD7 in the development of the glioma, we used the SETD7 siRNA to perform the knockdown experiment (Fig. S7k, l). The results showed that knockdown of SETD7 inhibited the proliferation and the AKT pathway of glioma (Figs. 5c–k, S10a, b). Moreover, our results revealed that the effects of upregulation of miR-1298-5p on proliferation were attenuated by overexpression of SETD7 (Fig. 6).

**Fig. 7:**
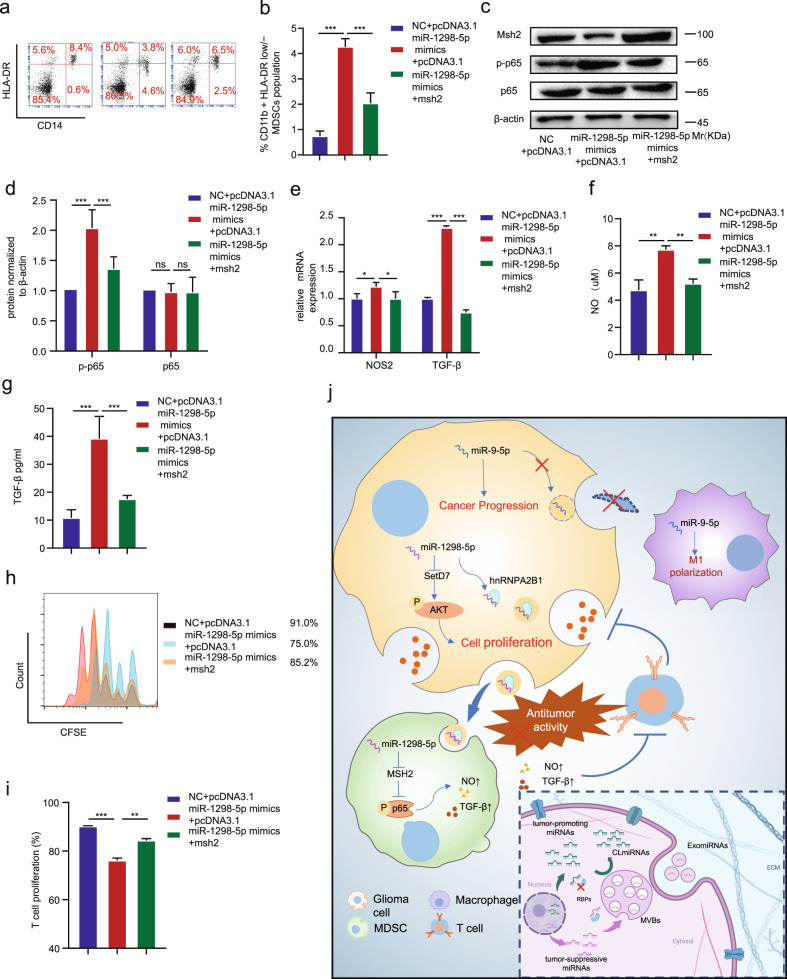
The effects of miR-1298-5p overexpression in MDSCs could be partially attenuated by MSH2 overexpression. **a**, **b** MDSCs transfected with miR-1298-5p mimics and pcDNA3.1-MSH2 or pcDNA3.1 were assessed by Flow cytometry assay. **c**, **d** The protein level of MSH2, p-p65 and p65 in MDSCs treated as described above were assessed by western blotting. Amounts of protein determined by densitometry of protein bands from three experiments. β-actin was the loading control. **e** The qRT-PCR assay showed the change of the expression of NOS2 and TGF-β in MDSCs treated as described above. **f**, **g** NO and TGF-β in the supernatants of MDSCs treated as described above. **h**, **i** CD8 + T cell proliferation was determined by flow cytometry 3 days later with CFSE dilution. **j** Schematic model showing that glioma selectively sorted oncosuppressor miR-1298-5p into exosomes and exosomal miR-1298-5p could promote the Immunosuppressive effects on of MDSCs. Moreover, miR-9-5p could promote glioma progression and induce M1 polarization of macrophages. Therefore, miR-9-5p was trapped inside cells. Data are shown as the mean ± SD of three independent experiments. Statistical significance was determined using one-way ANOVA test (*P < 0.05; **P < 0.01; ***P < 0.001).

To identify the targets of miR-1298-5p in MDSCs, we used the previous result predicted by the online bioinformatic tools. Among them, MSH2 has been reported to be associated with the NF-κB signaling pathway [20]. Therefore, MSH2 was hypothesized to be a direct target of miR-1298-5p and responsible for MDSCs activation. The qRT-PCR and western blot assay were used to determine that MSH2 was downregulated by miR-1298-5p (Fig. S7m, n). To confirm the binding site of miR-1298-5p in the 3’UTR of MSH2, we performed a luciferase reporter assay. And miR-1298-5p decreased the luciferase activity of the WT plasmid and didn’t affect the MUT plasmid (Fig. 5l, m). To explore the function of MSH2 in MDSCs, we knocked down MSH2 by using small interfering RNAs in MDSCs (Figs. S7o). The flow cytometry results showed that knocking down MSH2 increased the proportion of CD14^+^ HLA-DR^low/−^ MDSCs population (Figs. 5n, S10c). The qRT-PCR results demonstrated that MSH2 knockdown significantly increased the expression of NOS2 and TGF-βmRNA (Fig. S7p, q). Next, we measured NO and TGF-β production in MDSCs supernatants and got a consistent trend (Fig. 6o, p). The T cell-suppressive effect of MDSCs was then detected and MSH2-knockdown MDSCs suppressed T cell proliferation more strongly than the control group (Fig. 5q, r). In addition, MSH2 knockdown also affected the NF-κB pathway (Fig. 5s, t). Moreover, the effects of miR-1298-5p overexpression could be partially attenuated by MSH2 overexpression (Fig. 7).

In conclusion, our results showed that miR-1298-5p targeted setD7 in glioma and MSH2 in MDSCs.

### miR-9-5p could promote the progression of glioma and induce the M1 macrophage polarization

miR-9-5p was reduced in exosomes. Therefore, we hypothesized that miR-9-5p has the opposite effect of miR-1298-5p. To investigate the biological function of miR-9-5p in glioma, we overexpressed miR-9-5p in U87MG, U251. The transwell chamber assays and wound-healing assay showed that miR-9-5p could promote the migratory and invasive ability of glioma cells (Fig. S11a–d). Similarly, EdU assay and CCK8 assay were performed to examine the effects of miR-1298-5p on cell proliferation (Fig. S11e–g). qRT-PCR was performed to examine the expression of CD163, IL-10, IL-6, and TNF-α. Compared with miR-NC mimics, miR-9-5p could significantly increase the expression of IL-6 and TNF-α, and markedly decrease the expression of CD163 and IL-10 (Fig. S11h).

In conclusion, miR-9-5p could promote the progression of glioma and induce the M1 macrophage polarization.

### Immunosuppressive phenotype switch

To investigate the effect of sEV miRNAs in the glioma immune microenvironment, we further transfected the top 22 CSF sEV miRNAs into macrophages and then analyzed the flow cytometry data of immunosuppressive markers CD206. Our data indicated that almost all miRNAs could induce the M2 polarization of macrophages (Fig. S12). Herein, we believed that glioma cells could modulate the myeloid cells in the tumor microenvironment using sEV tumor-suppressor miRNAs that were not needed by tumor cells to produce a tumor-promoting phenotype transformation.

## Discussion

Accumulating evidence has proved that miRNAs play an essential role in tumor origin and progression [21]. The immunosuppressive tumor microenvironment is a complex system closely related to tumor progression [22]. Our previous study found that glioma exosomes could promote glioma-associated macrophage infiltration and M2 polarization and induce the expansion and immunosuppressive function of MDSCs [12, 23]. In our research, we reported the different effects of miR-1298-5p on glioma cells and MDSCs, respectively. We performed whole-transcriptome sequencing on CSF exosomes and matched glioma tissues. And we found some high-abundant tumor-suppressive miRNAs, such as miR-1298-5p, miR-122-5p, and miR-204-5p, showed an absolutely excretion tendency to sEV and help the tumor to resist antitumor immune. Some high-abundant tumor-promoting miRNAs such as miR-9-5p were almost stuck in tumor cells.

It has been reported that selective sorting of tumor-suppressor miRNA into exosomes promotes tumor progression [23, 24]. Similarly, we found that miR-1298-5p could target SETD7 to affect AKT pathway to inhibit tumor proliferation. It has been reported that SETD7 plays a critical role in hepatocellular carcinoma, colorectal cancer, lung cancer, and so on [25–28]. In this study, we found that SETD7 knockdown inhibited the proliferation of glioma via AKT pathway. PI3K–AKT signaling network plays an important role in supporting cancer survival and proliferation in various ways [29]. Considering its vital role in glioma proliferation, finding new strategies to deactivate the AKT system is essential to improving clinical outcomes for glioma patients.

Although it has been reported that tumors can sort oncosuppressor miRNA into exosomes to promote tumor progression, the effect of miRNA packaged into exosomes on TME is not clear. We hypothesized that miR-1298-5p in exosomes could resist anticancer immune activity. For the first time, we reported that miR-1298-5p targets the MSH2 gene and further potentiates MDSCs by activating the NF-κB pathway. We transfected glioma cells with Cy3-miR-1298-5p and cocultured them with MDSCs. Finally, Cy3-miR-1298-5p was detected in MDSCs, which demonstrated that glioma cells could transfer miR-1298-5p to MDSCs via exosomes. NF-κB pathway has been proved to activate MDSCs to resist anticancer immune activity [30]. We found miR-1298-5p activated NF-κB pathway in MDSCs. Therefore, preventing the transport of miR-1298-5p to exosomes is an effective way to weaken the immunosuppressive ability of MDSCs.

Exosomes could regulate the biological functions of recipient cells via miRNAs transfer. The exosomal RNAs are selectively loaded into exosomes by different RBPs [31]. It has been reported that hnRNPA2B1 regulates the sorting of miRNA into exosomes by binding to specific motifs [16]. In our research, we preformed RIP and RNA pull-down assay and found that hnRNPA2B1 binds to UUCA of miR-1298-5p and directs its packaging into exosomes. We knocked down hnRNPA2B1 in glioma cells and found that the exo/cell ratio of miR-1298-5p decreased. Moreover, we knocked down hnRNPA2B1 in glioma cells and cocultured them with MDSCs. And we found that the proportion of CD14^+^ HLA-DR ^low/-^ MDSCs population was decreased. These results may provide unique strategies for eliminating miR-1298-5p in the TME.

In summary, our findings reveal that miR-1298-5p plays a different role in glioma cells and MDSCs, respectively. It provided a new mechanism that glioma secreted oncosuppressor miR-1298-5p via exosomes to resist anticancer immune activity to acquire dual benefits. Other tumor-suppressive miRNAs are being researched in our research group, and we got the same result. Moreover, miR-1298-5p was enriched in exosomes of glioma patients, which can be used as a maker to achieve glioma liquid biopsy. In the future, nanoparticles or engineered extracellular vesicles can be used to knockdown hnRNPA2B1 targeting glioma cells to block the process. It will provide a theoretical basis for glioma treatment.

## Materials and methods

### Patients and data collection

Our study included CSF and tumor samples from 44 patients who were treated for glioma at Qilu Hospital of Shandong University from November 2017 to October 2019. The whole-course CSF sEV samples collected preoperatively were defined as ‘pre’, and the post-operative CSF samples were defined as ‘p1’ to ‘p6’ according to the collecting series. The 12 cases of non-tumorous brain tissues were obtained from the cortex of decompressive surgery patients with brain trauma or hypertensive intracerebral hemorrhage between November 2018 and April 2019 from the Department of Neurosurgery of the Qilu Hospital of Shandong University, the Second Hospital of Shandong University, and the 5th People’s Hospital of Jinan Shandong University. And 3 normal CSF (nor) were obtained from shunt procedures of normal pressure hydrocephalus (NPH) patients between February 2018 and December 2018 from the Department of Neurosurgery of the Qilu Hospital of Shandong University. All glioma patients had received surgical treatment microscopically. All patients authorized the informed consent, and this study was conducted in accordance with institutional ethical standards, the Declaration of Helsinki, and national and international guidelines. Ethics approval was obtained from the Clinical Research Ethics Committee of Qilu Hospital Shandong University.

### Magnetic resonance imaging

All patients underwent brain MRIs as indicated by the standard of care with standard sequences including axial T1-weighted, T2-weighted FLAIR, and contrast T1-weighted images. Brain MRIs were reviewed by an experienced neuroradiologist without knowledge of the CSF sEV sequencing results. The tumor regions were semi-automatically segmented slice by slice using 3D Slicer (www.slicer.org) on FLAIR and contrast T1-weighted sequence. To truncate outlier intensities, bias correction was applied on FLAIR and contrast T1-weighted sequence images to compensate for intensity non-uniformities using N4 algorithm [32] implemented in “extrantsr” package and then the intensities of images were normalized using “WhiteStripe” package in R which conducts white stripe normalization procedure [33]. Tumor region registration was performed by using “lesymap” package. The anatomical image of the patient was registered to the same geometric space as the template, the ICBM 152 2009c Nonlinear atlas, with the tumor regions being excluded. The transformation matrices obtained from the above registration were applied to the tumor region, and the tumor region was brought into template space. The tumor burden (sum of the products of the diameters, SPD), radiographic progression, and presence or absence of radiographic signs of tumor spread to subependymal, pial and leptomeningeal sites (CSF type) was according to the previous publications [34].

### Isolation and characterization of sEV from CSF

sEVs were isolated from CSF by differential centrifugation, according to the previous publications [35]. After removing cells and other debris by centrifugation at 2000 × *g* for 30 min, the supernatant was centrifuged at 12,000 × *g* for 45 min to remove shedding vesicles and the other vesicles with bigger sizes. The supernatant was centrifuged at 110,000 × *g* for 70 min and resuspended in 10 mL PBS. Finally, the suspension was recentrifuged at 110,000 × *g* for 70 min (all steps were performed at 4 °C); sEV were collected and resuspended in 50 μL PBS for further trans-omics RNA sequencing. The transmission electron microscopy assay of CSF sEV pellet was examined and photographed with an FEI Tecnai spirit TEM T12(FEI Tecnai Spirit 120 kv), according to the previous publications [35].

### RNA library preparation and sequencing

Total RNA from tissues and sEV of CSFs was isolated by using TRIzol Reagent (Invitrogen) according to the manufacturer’s instructions. RNA quality and quantity were assessed using a Nanodrop 2000 spectrophotometer (Thermo Fisher Scientific) and Agilent 2100 bioanalyzer (Agilent Technologies). Short-chain RNAs (miRNAs) and long-chain RNAs (mRNAs, lncRNAs, and circRNAs) libraries were prepared by using NEBNext® Multiplex Small RNA Library Prep Set for Illumina® (NEB) and NEBNext® UltraTM RNA Library Prep Kit (NEB), respectively. Long-chain RNA and miRNA sequencing was separately performed using the HiSeqX and HiSeq2500 platform (Illumina) according to the Illumina standard protocol by Beijing Novel Bioinformatics Co., Ltd. (https://en.novogene.com/).

### Quantification of transcripts abundance

Clean reads were obtained after the removal of reads containing adapters, reads containing ploy-N and low-quality reads from the raw Illumina sequencing reads. For long-chain RNAs, human reference genome and annotation files were downloaded from the genome website (NCBI/UCSC/Ensembl). Then, clean reads were aligned against the reference genome using HISAT2. To quantify the gene expression level, HTSeq was used to count the read numbers mapped for each gene. For miRNAs, clean reads were aligned against the human reference database (miRbase, http://www.mirbase.org/) using Bowtie and exact matches to known mature miRNA sequences in miRBase were counted. The Transcripts Per Million (TPM) of each miRNA was calculated based on the miRNA read counts. CIRI2 and Find_circ, the circRNA identification algorithms, were employed to predict circRNAs.

### Differential expression analysis

Normalization and differential expression analysis between glioma and control tissues as well as in sEV between preoperative (pre) and control CSF (p1 and nor) were performed by using the DEseq2 R package. The *P*-value was calculated and corrected for multiple testing using the Benjamini-Hochberg method. Normalized expression boxplots and volcano plots were generated by ggplot2 and ggpubr R package. Sample clustering, principal components analysis (PCA), hierarchical clustering were performed by using hcluster, prcomp, and pheatmap R package, respectively.

### The miRNA target prediction

Based on the differentially expressed mRNAs, lncRNAs, and circRNAs between glioma and control tissues, target mRNA, lncRNA, and circRNA of differentially expressed miRNAs in sEV of preoperative CSF (pre) and glioma tissue were obtained by the following steps. The target mRNAs were obtained using Targetscan (http://www.targetscan.org/), miRDB (http://www.mirdb.org/), miRTarBase (http://mirtarbase.mbc.nctu.edu.tw/) which were further filtered by Pearson Correlation Coefficient (PCC) analysis between the expression levels of mRNA and miRNA in glioma tissues (PCC < −0.3 and *P* < 0.05); the target lncRNAs were obtained by DIANA-LncBasev2.0 (http://carolina.imis.athena-innovation.gr/diana_tools/web/index.php?r = lncbasev2/index-predicted) and StarBase 2.0 (http://starbase.sysu.edu.cn/starbase2/index.php); the target circRNAs were obtained by miRDB (http://www.mirdb.org/) and StarBase 2.0 (http://starbase.sysu.edu.cn/starbase2/index.php). Finally, a competitive endogenous RNA (ceRNA) network was constructed by using Cytoscape 3.7.0 software after the PCC analysis of expression levels between lncRNA and mRNA (PCC > 0.3 and *P* < 0.05) as well as circRNA and mRNA (PCC > 0.3 and *P* < 0.05) in glioma tissues.

### Pathway enrichment analysis

Pathway enrichment analysis was done on target genes of up and downregulated miRNAs in preoperative CSF sEV (pre) compared with glioma tissue using the clusterProfiler R package. Firstly, symbol gene IDs were converted to Entrez gene IDs. Then, Kyoto Encyclopedia of Genes and Genomes (KEGG) enrichment analysis was implemented.

### Cell culture

P3 cell line was kindly provided by Prof. Rolf Bjerkvig, University of Bergen, which was isolated from human glioblastoma tissue. P3 cells were maintained in neurobasal medium (NBM) supplemented with GlutaMAX (2 mM), B-27(1×), penicillin/streptomycin (1×), heparin(32 IE/ml), EGF(20 ng/ml), and FGF2(20 ng/ml). U87MG, U251, A172 (Chinese Academy of Sciences Cell Bank), and LN229 cells (ATCC) were cultured in DMEM (Sigma) supplemented with 10% FBS (Thermo Fisher Scientific). All the cell lines were incubated at 37 °C with 5% CO_2_ and 95% air. All cells were authenticated by short tandem repeat (STR) profiling and routinely tested for mycoplasma contamination.

### Peripheral blood mononuclear cells (PBMC) and monocyte isolation

PBMCs were isolated from the venous blood of healthy donors and patients using a lymphocyte separation medium (LTS1077, TBD, China). Blood was separated using standard density gradient centrifugation (30 min at 500 × *g* at 21 °C), and the PBMC layer was carefully transferred to another tube. CD14 + monocytes were separated from PBMCs using CD14 MicroBeads (Miltenyi Biotec, Germany) according to the manufacturer’s protocol. Purity (> 95%) was confirmed by flow cytometry.

### MDSC induction

PBMCs (5 × 10 ^5^) from healthy donors were cultured in 12-well plates with 0.5 ml of exosome-depleted RPMI 1640 complete medium. CSF- or glioma cell-derived exosomes were added to the culture medium and cocultured with PBMCs for 72 h. PBMCs were examined via flow cytometry following the coculture.

### Exosomal miRNA transportation assay

Cy3-labeled miR-1298-5p was purchased from GenePharma. GDEs were isolated from the supernatant of U87MG and P3 cells transfected with Cy3-labeled miR-1298-5p and used to treat monocytes. Two days later, the monocytes were collected and stained with DAPI and FITC-phalloidin.

### Exosome uptake assay

Exosomes were labeled with PKH67 (Sigma–Aldrich, USA) according to the manufacturer’s protocol. PKH lipophilic dyes are fluorescent and their aliphatic domains intercalate into lipid bilayers, such that exosomes stained with PKH67 can be visualized via fluorescence microscopy. The PKH67-labeled exosomes were incubated with monocytes. Once the PKH67-labeled exosomes are internalized by monocytes, the fluorescence signal from PKH67 can be observed within the recipient cells. Briefly, exosomes were reconstituted in 50 µl PBS before 1 ml of Diluent C was added. Four microliters of PKH67 dye were added to 1 ml of Diluent C before being added to the exosomes. The samples were mixed gently for 4 min. To neutralize the excess dye, the PKH67-labeled exosome solution was mixed with 3 ml 0.5% BSA and centrifuged at 100,000 × *g* for 1 h. The exosome pellet was resuspended in PBS and added to the culture medium of human monocytes. The monocytes were then fixed and examined under a fluorescence microscope.

### T cell suppression assay

CD14-depleted PBMCs were labeled with 2.5 μM CFSE and stimulated with coated anti-CD3 (1 μg/ml, eBioscience) and soluble anti-CD28 (1 μg/ml, eBioscience) antibodies. Exosome-induced monocytes were cultured with these cells at a ratio of 1:2 in a U-bottom 96-well plate. Three days later, the cells were stained with anti-CD8-APC antibody and analyzed for CFSE dilution.

### Induction of M2 macrophages

PBMCs were isolated from the blood of healthy donors as previously described [9]. Blood was separated using standard density gradient centrifugation (30 min at 500 × *g* at 21 °C, LTS1077, TBD). PBMCs were extracted from the interphase. CD14 + cells were selected using magnetic CD14-positive beads (Miltenyi Biotec, 130-050-201). To differentiate these monocytes into monocyte-derived macrophages, CD14 + cells were cultured in RPMI 1640 media (Thermo Fisher Scientific) supplemented with 10% FBS and 100 ng/ml M-CSF (PeproTech). Six days later, the cells were cultured in 0.5 ml Opti-MEM containing 4 μl Lipofectamine3000 and 20pmol mimics. The Opti-MEM was replaced with complete RPMI 1640 medium 6 h later. Two days later, cells were stained with anti-CD206-APC (Invitrogen, 17-2069-42). Flow cytometry was performed using the Beckman Coulter Gallios and data were analyzed using FlowJo software.

### RNA extraction and quantitative reverse-transcription (qRT-PCR)

Total cell RNA was extracted using RNA-Quick Purification Kit (ESscience Biotech, China) according to the manufacturer’s protocol. ReverTra Ace qPCR RT Master Mix (Toyobo, Japan) was used to synthesize cDNA following the manufacturer’s instructions. qRT-PCR was performed with SYBR Green PCR Master Mix (Applied Biosystems, Foster City, USA). Expression data of microRNA and mRNA were normalized to the internal controls U6 and GAPDH, respectively. The relative expression levels were calculated using the ΔΔCt method. The sequence of primers is shown in Supplementary Table 6.

### Western blotting

Whole-cell protein was extracted from glioma cells and MDSCs in RIPA buffer (Thermo Fisher Scientific, USA) and centrifuged at 12,000 rpm for 20 min. A BCA kit (Thermo, Waltham, MA, 23228) was used to measure the protein concentration. After immunoblotting, the proteins were transferred to a nitrocellulose membrane and incubated with specific antibodies. The following primary antibodies were used: β-actin (Proteintech, 60008-1-Ig), CyclinD1 (Cell Signaling Technology, 2978), P27 (Cell Signaling Technology, 3686), CDK6 (Cell Signaling Technology, 3136), p-AKT (Cell Signaling Technology, 4060), AKT (Cell Signaling Technology, 4691), SETD7 (Cell Signaling Technology, 2813), hnRNPA2/B1 (Cell Signaling Technology, 9304), Phosphorylated NF-κB p65 (S536) (Cell Signaling Technology, 3033), and NF-κB p65 (Cell Signaling Technology, 8242).

### Small interfering RNA, miR mimics, and adenovirus vector transfection

Control microRNAs, miR-1298-5p mimics, were purchased from GenePharma (Shanghai, China). si-setD7, si-MSH2, and control siRNAs were purchased from RiboBio (Guangzhou, China). All sequences are listed in Supplementary Table 6. For microRNA and siRNA transfection, cells were incubated in six-well plates overnight and transfected with LipofectamineTM 3000 reagent (Thermo Fisher Scientific, USA) according to the manufacturer’s protocol. The miR-1298-5p overexpression and control lentiviruses were synthesized by Genechem (Shanghai, China). The knockdown/overexpression efficiency of the siRNAs and viruses is available in the supplementary materials.

### CCK8 assay

Cell Counting Kit-8 (CCK8) was used to measure cell viability according to the manufacturer’s instructions (Beyotime, China). Cells were seeded at 5000 cells per well into 96-well plates and cultured at 37 °C with different treatments. CCK8 solution (10 µl) was added at 24, 48, and 72 h. Following incubation for 2 h, the absorbance at 450 nm (OD450) was measured using a Multimode Plate Reader (PerkinElmer, USA).

### Cell cycle analysis

Glioma cells were stained with Propidium iodide (PI) in the presence of RNase A for 15 min. Flow cytometer (BD Biosciences) was used to perform cell cycle analysis according to the protocol.

### Ethynyl-2’-deoxyuridine (EdU) cell proliferation assay

EdU assay kit (Ribobio, China) was used to test the cell proliferation ability according to the manufacturer’s instructions. Glioma cells were seeded into wells of poly-l-ornithine precoated 12-well plates. Cells were then incubated with 200 μl of 5-ethynyl-20-deoxyuridine for 2 h at 37 °C. Nuclei were counterstained with Hoechst 33342. Representative images were obtained with a Leica inverted fluorescence microscope.

### Dual-luciferase reporter assay

The dual-luciferase reporter plasmids (SETD7 WT/MUT and MSH2 WT/MUT) were designed and synthesized by GenePharma (Shanghai, China). HEK-293T cells were seeded in 96-well plates overnight (2 × 104 / well). For 3’UTR tests, the dual-luciferase reporter plasmids (0.1 μg/ well) were co-transfected with miR-Nc and miR-1298-5p mimics (20 nM × 0.5 μl/well). Approximately 48 h after transfection, the cells were subjected to luciferase activity analysis using a Dual-Luciferase Reporter Assay System (Promega) following the manufacturer’s instructions.

### Exosome isolation

Cells were cultured in DMEM supplemented with 10% exosome-depleted FBS under normoxic (21% O_2_) or hypoxic (1% O_2_) conditions. Exosomes were isolated from cell culture supernatant as previously described for later analysis.

### Electron microscopy and qNano

Isolated exosomes were examined using Transmission Electron Microscopy (TEM) as previously described. qNano (Izon Sciences Ltd, NZ) was used for exosome particle size and concentration analysis [18].

### Cytokine assay

Cell culture medium was collected 72 h after the indicated treatment. The secretion of TNF-β was detected by ELISA (Proteintech, USA) and the NO was measured using Greiss regent, according to the manufacturer’s instructions.

### RNA binding protein immunoprecipitation

The RIP assays were performed using an EZ-Magna RIP kit (Millipore). Lysates of 1 × 107 glioma cells obtained using complete RIP lysis buffer were immunoprecipitated with RIP buffer containing anti-hnRNPA2B1 antibody-conjugated magnetic beads (Abcam). The precipitated RNAs were analyzed by qRT-PCR. Mouse IgG was used as the negative control.

### RNA pull-down assay

The miR-1298-5p-binding proteins were examined using RNA pull-down assays according to the instructions of the Pierce Magnetic RNA-Protein Pull-Down Kit (ThermoFisher Scientific). Biotinylated miR-1298-5p and control sequences were synthesized by GenePharma (Shanghai, China). The cell lysate obtained using a Pierce IP Lysis Buffer (Thermo Fisher Scientific) was incubated overnight with biotinylated miR-1298-5p, followed by precipitation with streptavidin magnetic beads. The retrieved protein was eluted from the RNA-protein complex and analyzed by western blotting.

### Animal studies

Luciferase-labeled and stably transfected U87MG cells overexpressing miR-1298-5p or vector were injected into the brains of randomly grouped 4-week BALB/c nude mice (5 × 10 5/mouse) to build the orthotopic xenograft model. Bioluminescence imaging was used to image the mouse brains every 5 days after glioma cell implantation. Next, we randomly chose five mice in each group and euthanized them on the same day (10 d). The brains were fixed with paraformaldehyde for further study. The remaining mice (5/group) were kept until death for survival analysis. All procedures that involved mice were approved by and under the requirements of the Animal Care and Use Committee of the Qilu Hospital of Shandong University.

### Statistical analysis

The cut-off value between high and low miR-1298-5p expression was set as the expression level of a median sample. Survival analysis was performed using the Kaplan–Meier method and comparisons were done using the log-rank test. The one-way ANOVA test or Student’s *t*-test was used for all other data comparisons using GraphPad Prism 8. All data are presented as the mean ± standard error and *P*-values <0.05 were considered statistically significant.

## Supplementary information

supplementary figures

AJ Checklist

Table S1

Table S2

Table S3

Table S4

Table S5

Table S6

uncropped westernblots

## Data Availability

The datasets used and/or analyzed during the current study are available in Supplementary Tables and the full original data are available from National Genomics Data Center (GSA: CRA002339) or the corresponding author on reasonable request.

## References

[1] Reifenberger G, Wirsching HG, Knobbe-Thomsen CB, Weller M (2017). Advances in the molecular genetics of gliomas—implications for classification and therapy. Nat Rev Clin Oncol.

[2] Lim M, Xia Y, Bettegowda C, Weller M (2018). Current state of immunotherapy for glioblastoma. Nat Rev Clin Oncol.

[3] Galon J, Bruni D. Tumor immunology and tumor evolution: intertwined histories. Immunity. 2020;52:55–81. 10.1016/j.immuni.2019.12.018 31940273

[4] Quail D, Joyce JJCC (2017). The microenvironmental landscape of brain tumors. Cancer Cell..

[5] Sampson J, Gunn M, Fecci P, Ashley DJNRC (2020). Brain immunology and immunotherapy in brain tumours. Nat Rev Cancer..

[6] Hoshino A, Kim HS, Bojmar L, Gyan KE, Cioffi M, Hernandez J (2020). Extracellular vesicle and particle biomarkers define multiple human cancers. Cell..

[7] An T, Qin S, Xu Y, Tang Y, Huang Y, Situ B (2015). Exosomes serve as tumour markers for personalized diagnostics owing to their important role in cancer metastasis. J Extracell Vesicles..

[8] Su T, Zhang P, Zhao F, Zhang SJFIO (2021). Exosomal microRNAs mediating crosstalk between cancer cells with cancer-associated fibroblasts and tumor-associated macrophages in the tumor microenvironment. Front Oncol.

[9] Guo X, Xue H, Shao Q, Wang J, Guo X, Chen X (2016). Hypoxia promotes glioma-associated macrophage infiltration via periostin and subsequent M2 polarization by upregulating TGF-beta and M-CSFR. Oncotarget..

[10] Guo X, Qiu W, Liu Q, Qian M, Wang S, Zhang Z (2018). Immunosuppressive effects of hypoxia-induced glioma exosomes through myeloid-derived suppressor cells via the miR-10a/Rora and miR-21/Pten. Pathways..

[11] Guo X, Qiu W, Wang J, Liu Q, Qian M, Wang S (2019). Glioma exosomes mediate the expansion and function of myeloid-derived suppressor cells through microRNA-29a/Hbp1 and microRNA-92a/Prkar1a pathways. Int J Cancer..

[12] Qian M, Wang S, Guo X, Wang J, Zhang Z, Qiu W (2019). Hypoxic glioma-derived exosomes deliver microRNA-1246 to induce M2 macrophage polarization by targeting TERF2IP via the STAT3 and NF-κB pathways. Oncogene..

[13] Bronisz A, Rooj A, Krawczyński K, Peruzzi P, Salińska E, Nakano I (2020). The nuclear DICER-circular RNA complex drives the deregulation of the glioblastoma cell microRNAome. Sci Adv..

[14] Guo X, Qiu W, Liu Q, Qian M, Wang S, Zhang Z (2018). Immunosuppressive effects of hypoxia-induced glioma exosomes through myeloid-derived suppressor cells via the miR-10a/Rora and miR-21/Pten Pathways. Oncogene..

[15] Zhang X, Yuan S, Li H, Zhan J, Wang F, Fan J (2021). The double face of miR-320: cardiomyocytes-derived miR-320 deteriorated while fibroblasts-derived miR-320 protected against heart failure induced by transverse aortic constriction. Signal Transduct Target Ther..

[16] Chen C, Luo Y, He W, Zhao Y, Kong Y, Liu H (2020). Exosomal long noncoding RNA LNMAT2 promotes lymphatic metastasis in bladder cancer. J Clin Invest.

[17] Hu B, Yang YT, Huang Y, Zhu Y, Lu ZJ (2017). POSTAR: a platform for exploring post-transcriptional regulation coordinated by RNA-binding proteins. Nucleic Acids Res.

[18] Li J-H, Liu S, Zhou H, Qu L-H, Yang J-H (2014). starBase v2.0: decoding miRNA-ceRNA, miRNA-ncRNA and protein–RNA interaction networks from large-scale CLIP-Seq data. Nucleic Acids Res.

[19] Wong N, Wang X (2015). miRDB: an online resource for microRNA target prediction and functional annotations. Nucleic Acids Res.

[20] Eso Y, Takai A, Matsumoto T, Inuzuka T, Horie T, Ono K (2016). MSH2 dysregulation is triggered by proinflammatory cytokine stimulation and is associated with liver cancer development. Cancer Res.

[21] Wang LQ, Yu P, Li B, Guo YH, Liang ZR, Zheng LL (2018). miR‐372 and miR‐373 enhance the stemness of colorectal cancer cells by repressing differentiation signaling pathways. Mol Oncol.

[22] Quail DF, Joyce JA (2017). The microenvironmental landscape of brain tumors. Cancer Cell.

[23] Teng Y, Ren Y, Hu X, Mu J, Samykutty A, Zhuang X (2017). MVP-mediated exosomal sorting of miR-193a promotes colon cancer progression. Nat Commun.

[24] Santangelo L, Giurato G, Cicchini C, Montaldo C, Mancone C, Tarallo R (2016). The RNA-binding protein SYNCRIP is a component of the hepatocyte exosomal machinery controlling microRNA sorting. Cell Rep..

[25] Cao L, Ren Y, Guo X, Wang L, Zhang Q, Li X (2020). Downregulation of SETD7 promotes migration and invasion of lung cancer cells via JAK2/STAT3 pathway. Int J Mol Med..

[26] Chen Y, Yang S, Hu J, Yu C, He M, Cai Z (2016). Increased expression of SETD7 promotes cell proliferation by regulating cell cycle and indicates poor prognosis in hepatocellular carcinoma. PLoS ONE.

[27] Guo T, Wen XZ, Li ZY, Han HB, Zhang CG, Bai YH (2019). ISL1 predicts poor outcomes for patients with gastric cancer and drives tumor progression through binding to the ZEB1 promoter together with SETD7. Cell Death Dis.

[28] Duan B, Bai J, Qiu J, Wang J, Tong C, Wang X (2018). Histone-lysine N-methyltransferase SETD7 is a potential serum biomarker for colorectal cancer patients. EBioMedicine..

[29] Hoxhaj G, Manning BD (2020). The PI3K-AKT network at the interface of oncogenic signalling and cancer metabolism. Nat Rev Cancer.

[30] Porta C, Consonni FM, Morlacchi S, Sangaletti S, Bleve A, Totaro MG (2020). Tumor-derived prostaglandin E2 promotes p50 NF-kappaB-dependent differentiation of monocytic MDSCs. Cancer Res.

[31] Shurtleff MJ, Temoche-Diaz MM, Karfilis KV, Ri S, Schekman R. Y-box protein 1 is required to sort microRNAs into exosomes in cells and in a cell-free reaction. eLife. 2016;5:e19276. 10.7554/eLife.19276 PMC5047747 27559612

[32] Tustison N, Avants B, Cook P, Zheng Y, Egan A, Yushkevich P (2010). N4ITK: improved N3 bias correction. IEEE Trans Med Imaging..

[33] Shinohara R, Sweeney E, Goldsmith J, Shiee N, Mateen F, Calabresi P (2014). Statistical normalization techniques for magnetic resonance imaging. Neuroimage Clin.

[34] Miller A, Shah R, Pentsova E, Pourmaleki M, Briggs S, Distefano N (2019). Tracking tumour evolution in glioma through liquid biopsies of cerebrospinal fluid. Nature..

[35] Zhang H, Deng T, Liu R, Bai M, Zhou L, Wang X (2017). Exosome-delivered EGFR regulates liver microenvironment to promote gastric cancer liver metastasis. Nat Commun..

