# Supplementary material for: The dual role of glioma exosomal microRNAs: glioma eliminates tumor suppressor miR-1298-5p via exosomes to promote immunosuppressive effects of MDSCs
Source: Cell Death Dis. 2022 May 2;13(5):426. doi: 10.1038/s41419-022-04872-z (PMC9061735; doi:10.1038/s41419-022-04872-z)
Supplement: Supplementary file 1 — supplementary figures [file 41419_2022_4872_MOESM1_ESM.docx]

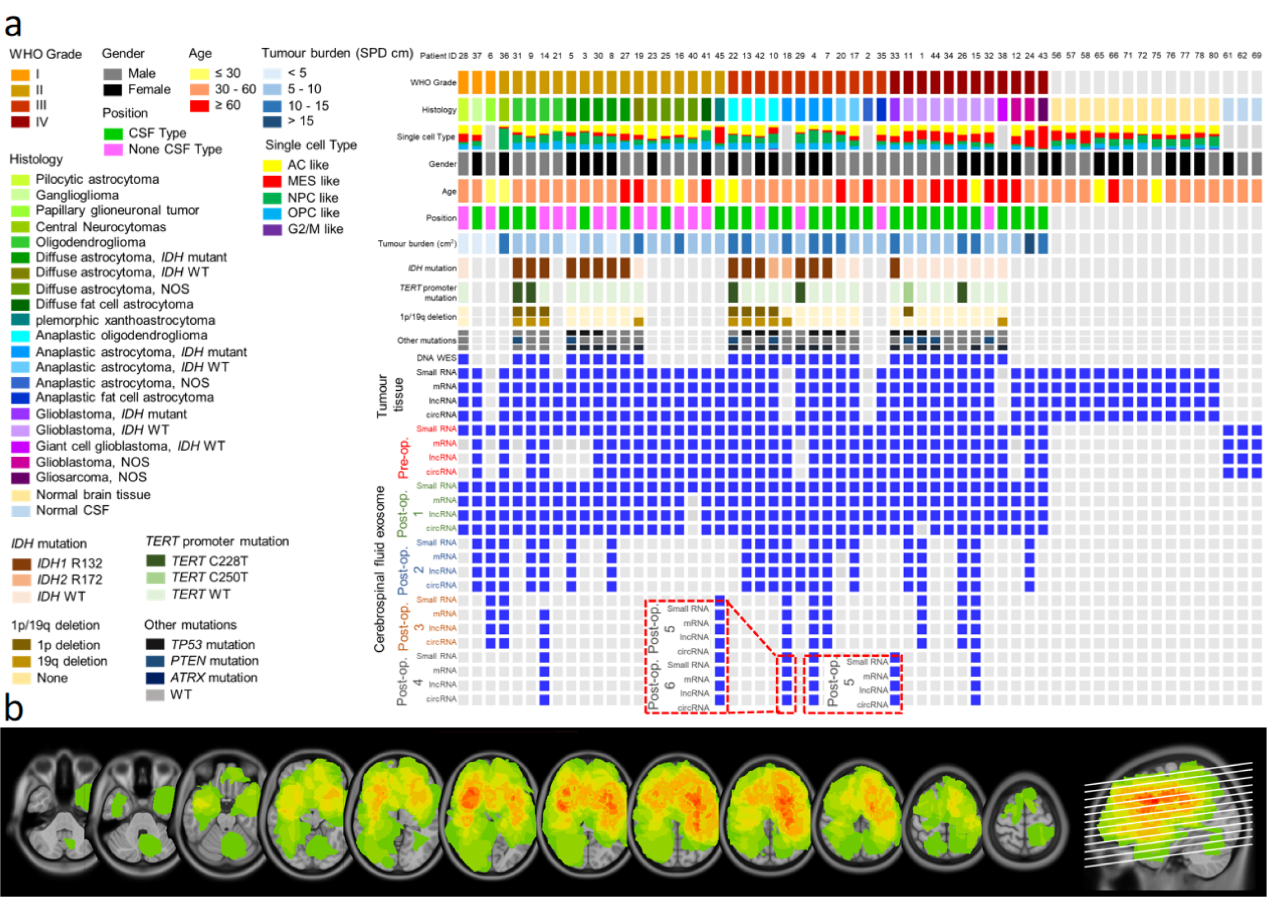
Fig. S1 CSF sEV RNA database of glioma cohort.

(a) Overview of the 44 glioma patients and 15 control with 12 normal brain tissues and three normal CSF of our cohort. Shown are the baseline characteristics, including WHO grade, histology, simulated single cell type, gender, age, position, tumor burden, and the most common genetic alterations. The blue squares below represent a panorama of all sequencing samples in the database. (b) The tumors' distribution locations in all 44 patients' brains were delineated by 3D Slicer (version 4.10.1).


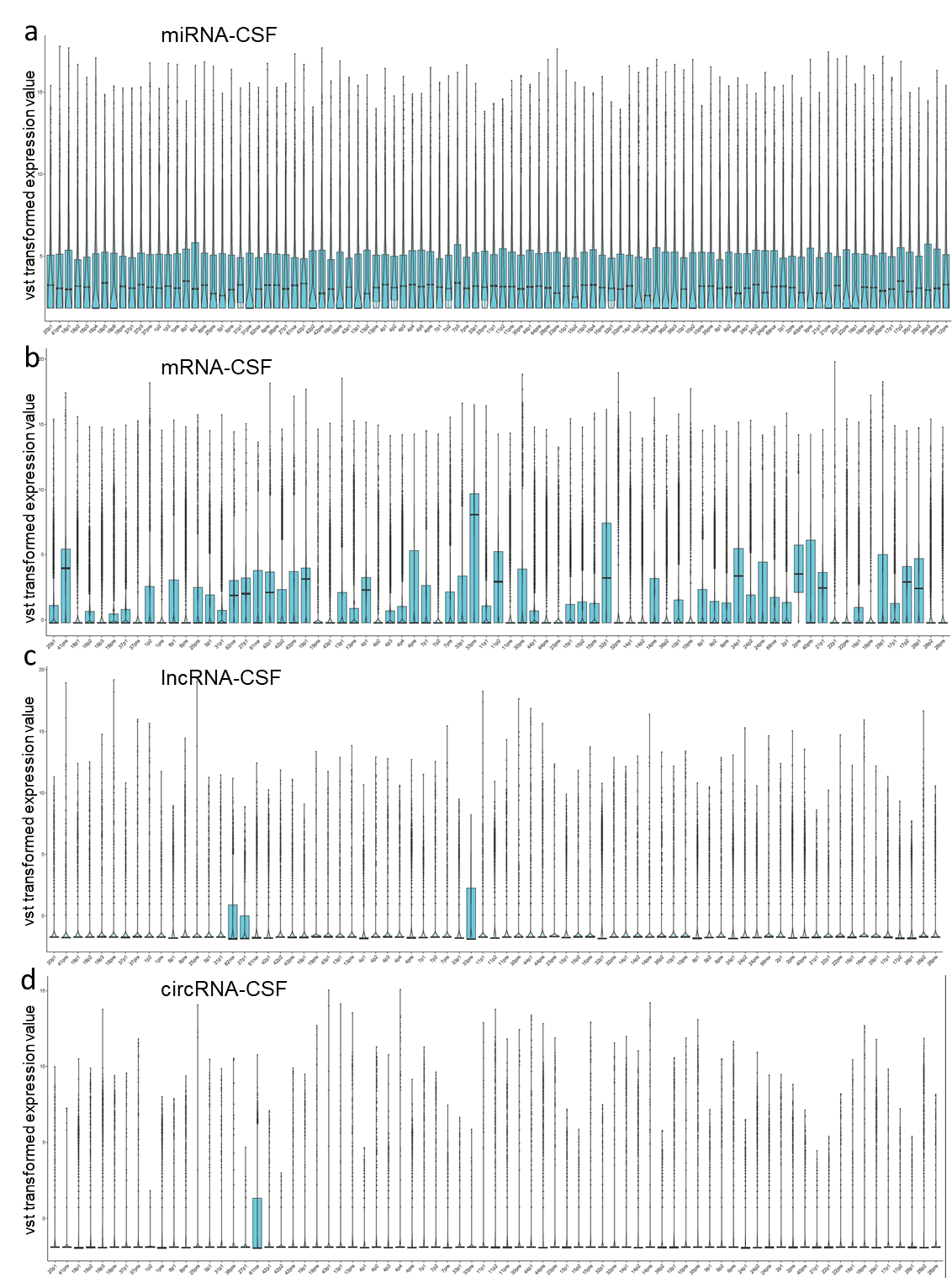


Fig. S2 Violin- and box-plot analysis comparing the levels and distributions of RNAs in sEV of CSF. The x-axis and y-axis represent samples and the VST (variance-stabilizing transformation)-transformed expression value from DESeq2 results, respectively. The median and quartile display on each violin-plot using box-plot. Only miRNAs (a) showed relatively high and stable expression and distribution characteristics in CSF sEV, while mRNAs (b), lncRNAs (c), and circRNAs (d) expression in CSF sEV were low and unstable.


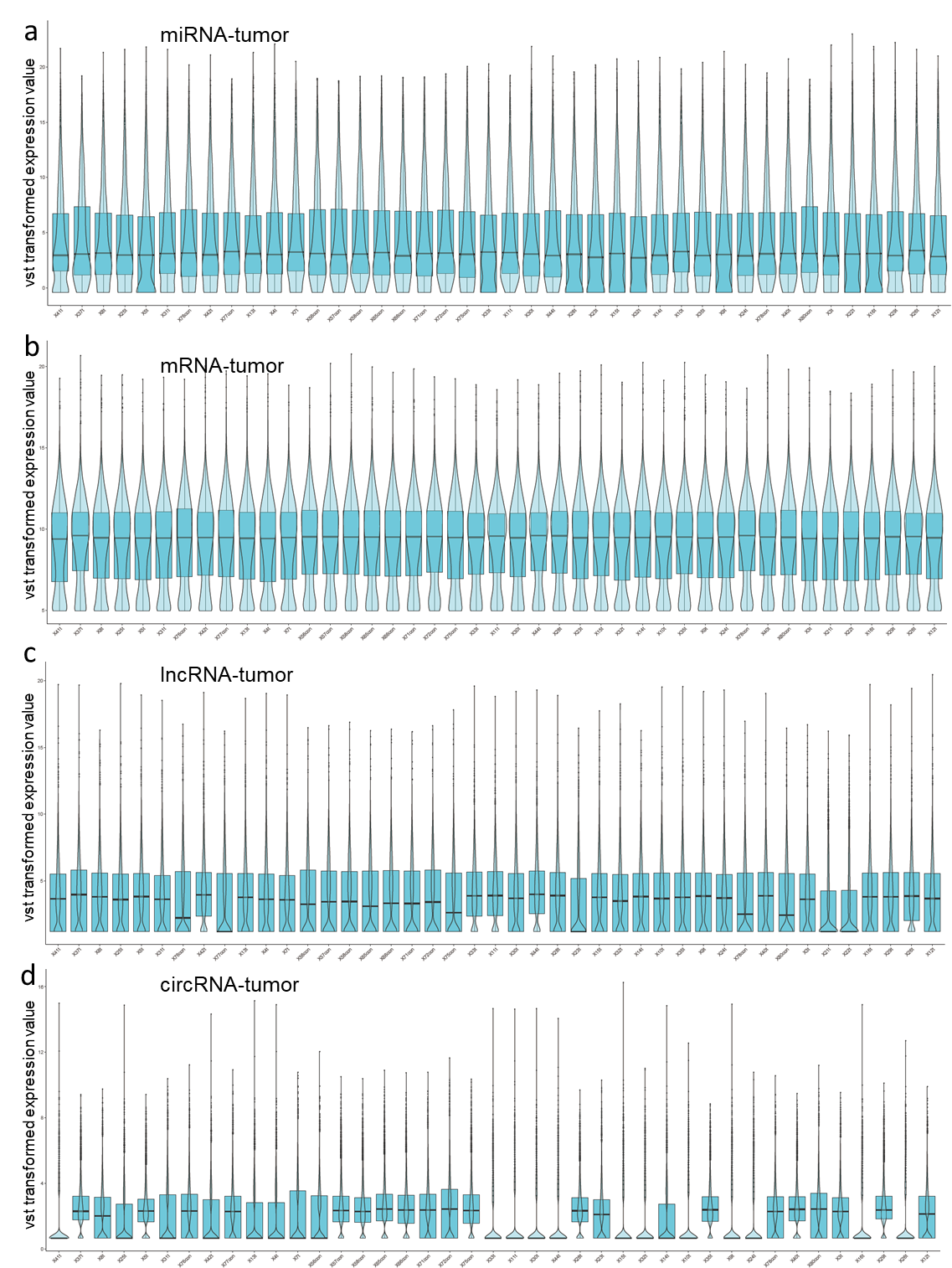


Fig. S3 Violin- and box-plot analysis comparing the levels and distributions of RNAs in tissues. The x-axis and y-axis represent samples and the VST (variance-stabilizing transformation)-transformed expression value from DESeq2 results, respectively. The median and quartile display on each violin-plot using box-plot. Except for circRNA (d), miRNAs (a), mRNAs (b) and lncRNAs (c) showed relatively high and stable expression and distribution characteristics in tissues, including glioma and control tissues.


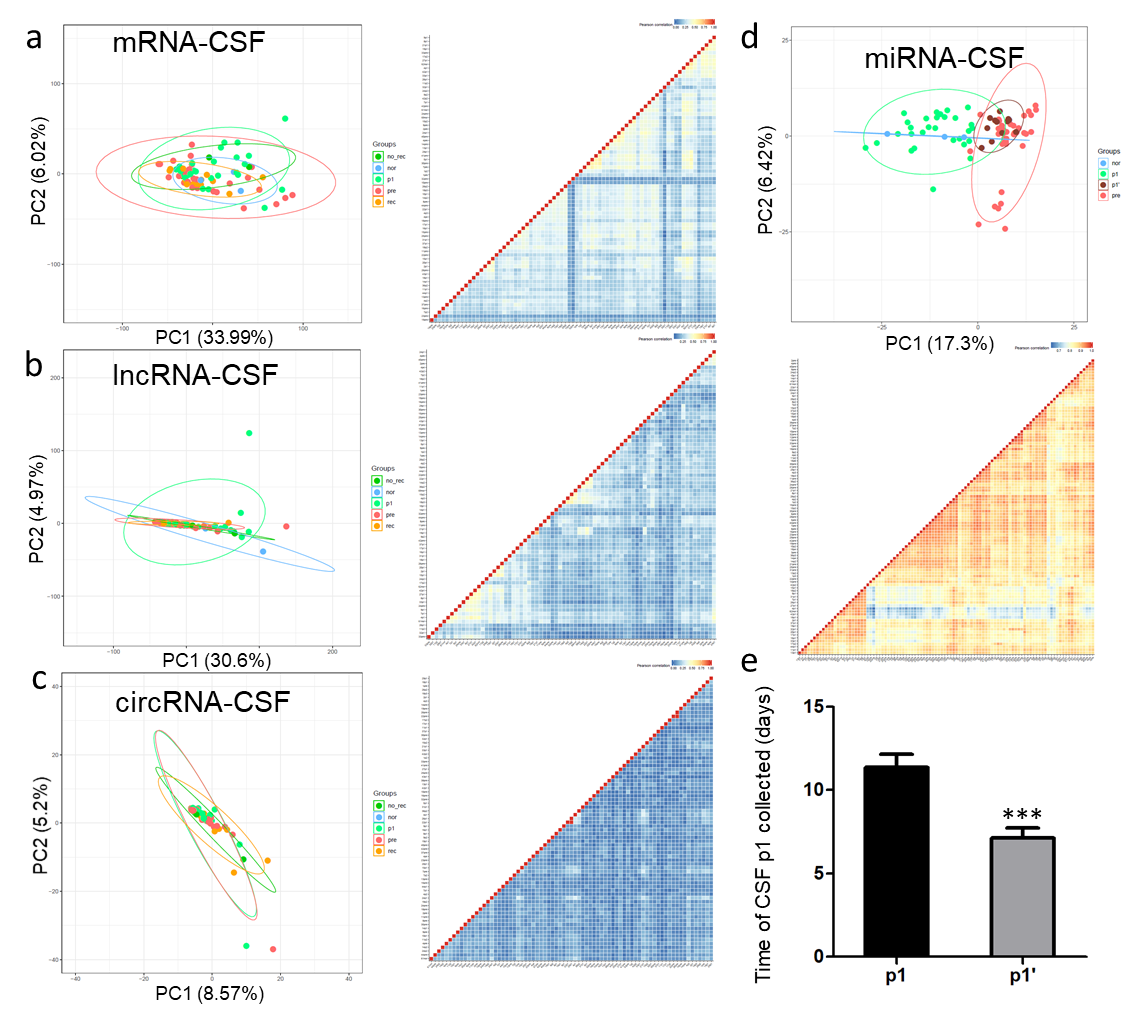
Fig. S4 CSF sEV mRNA, lncRNA and circRNA insufficiently indicating tumor-burdened status in glioma patients. a, b, c, The ability of tumor- burdened status discrimination of mRNA, lncRNA and circRNA in CSF sEV. Shown are the PCA score and distribution plots (left) of miRNA expression in pre-operation CSF sEV (pre), post-operation No.1 CSF sEV (p1), normal CSF sEV (nor) and sEV of the following up CSF samples with (rec) or without recurrence (no_rec) and the sample clustering (pairwise Pearson correlation Coefficient analysis) of all CSF sEV samples of our cohort (right). d, The misclassified post-operation CSF sEV miRNA samples (p1') were shown in the PCA score and distribution plots (up), and the sample clustering (down). e, The number of days from operation to the time of p1' CSF collection was significantly less than that of p1 CSF collection.


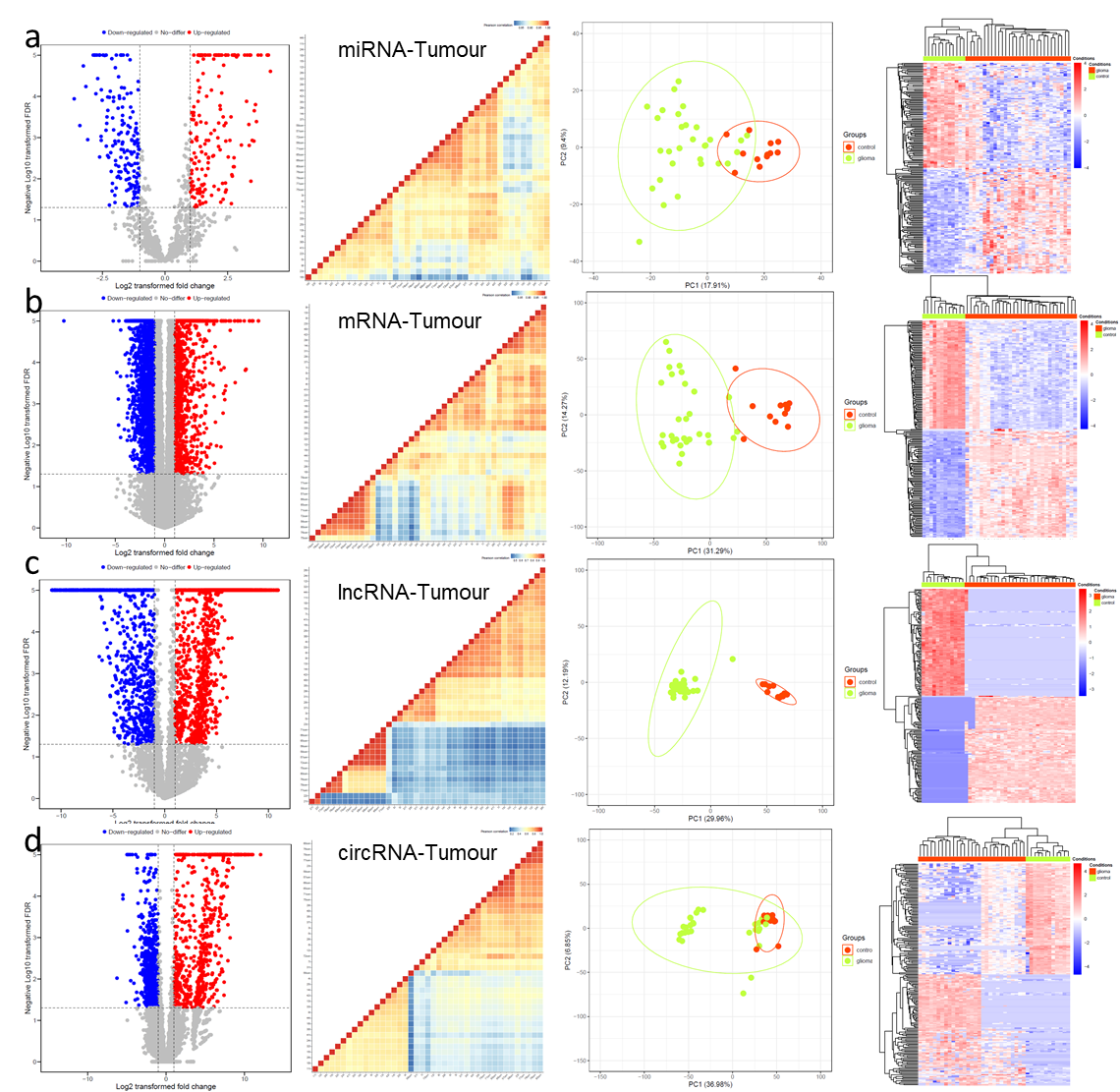
Fig. S5 Expression profile of miRNA, mRNA, lncRNA and circRNA faultlessly identifying glioma tissues from control brain tissues. The ability of tumor and normal brain tissue discrimination of miRNA (a), mRNA (b), lncRNA (c) and circRNA (d) in surgical specimens. The panels in order (from left to right) were volcano plot, sample clustering (Pearson Correlation Coefficient) and Principal Component Analysis of RNA expression levels and unsupervised hierarchical cluster analysis of the top 100 up- and down-regulated RNAs (|log2foldchange|≥1 and FDR < 0.05, sorted by FDR) in glioma tissues compared with control brain tissues.


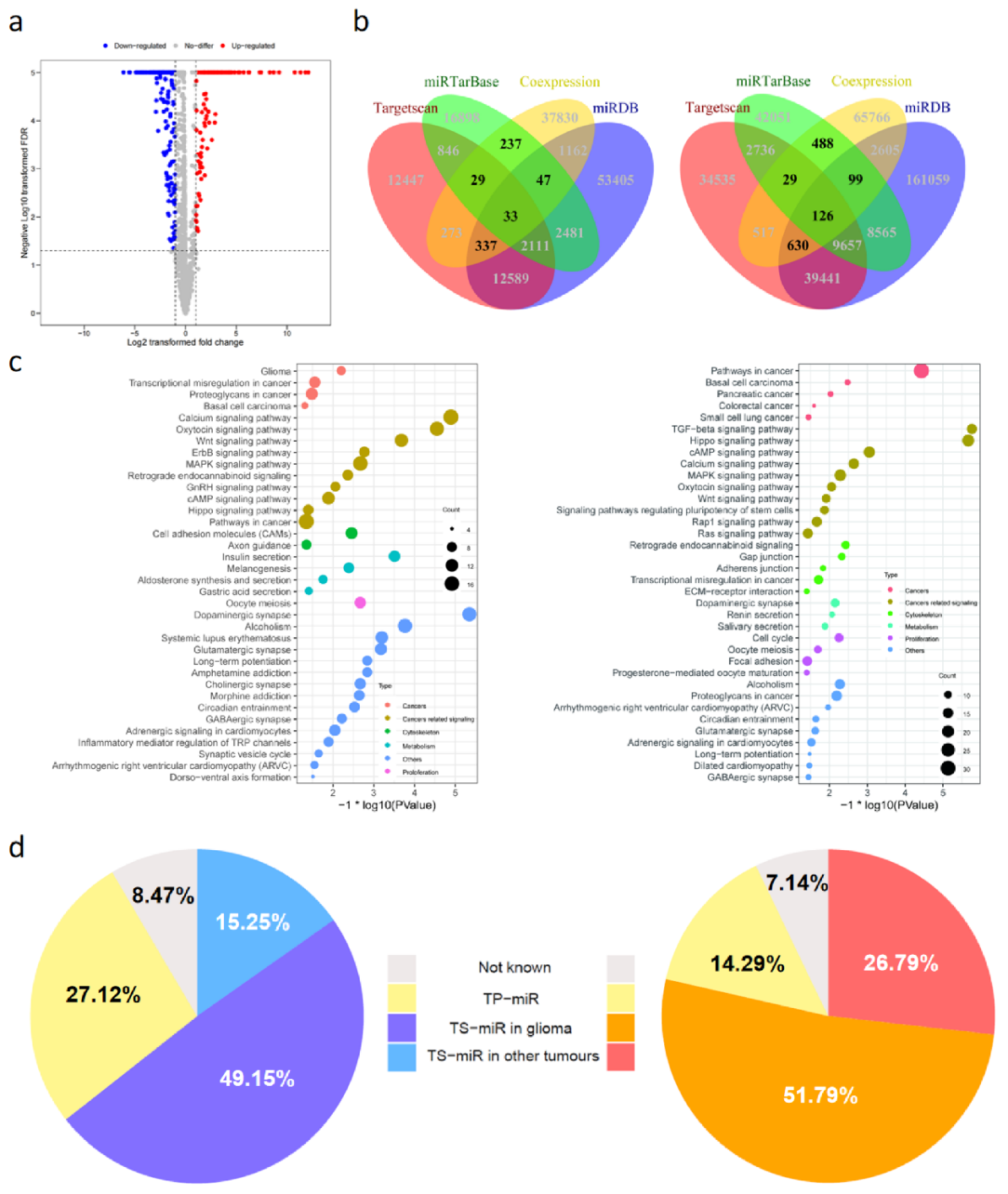


Fig. S6 Tumor suppressive miRNAs dominated CSF sEV of glioma patients.

1. The volcano plot of differentially expressed miRNAs between pre-operation CSF sEV and glioma tissues. (b) Venn diagram of the predicted target mRNAs of above-mentioned differential expressed miRNAs (up- and down- regulated, respectively). The red, green, purple and yellow circle separately represented the targets obtained using Targetscan, miRTarBase, miRDB and Pearson Correlation Coefficient (PCC) analysis between the expression levels of mRNA and miRNA in glioma tissues (PCC< -0.3 and p value<0.05). The selected target sets shown in black font were retained for pathway enrichment analysis. (c) KEGG pathway enrichment analysis for targets of above-mentioned differential expressed miRNAs (up- and down- regulated bubble chart, respectively). (d) Pie chart of high-expressed CSF sEV miRNAs indicated the proportion of tumor-suppressive miRNAs (TS-miR) in glioma and other tumors, tumor-promoting miRNAs (TP-miR), and function unknown miRNA (up- and down- regulated, respectively).


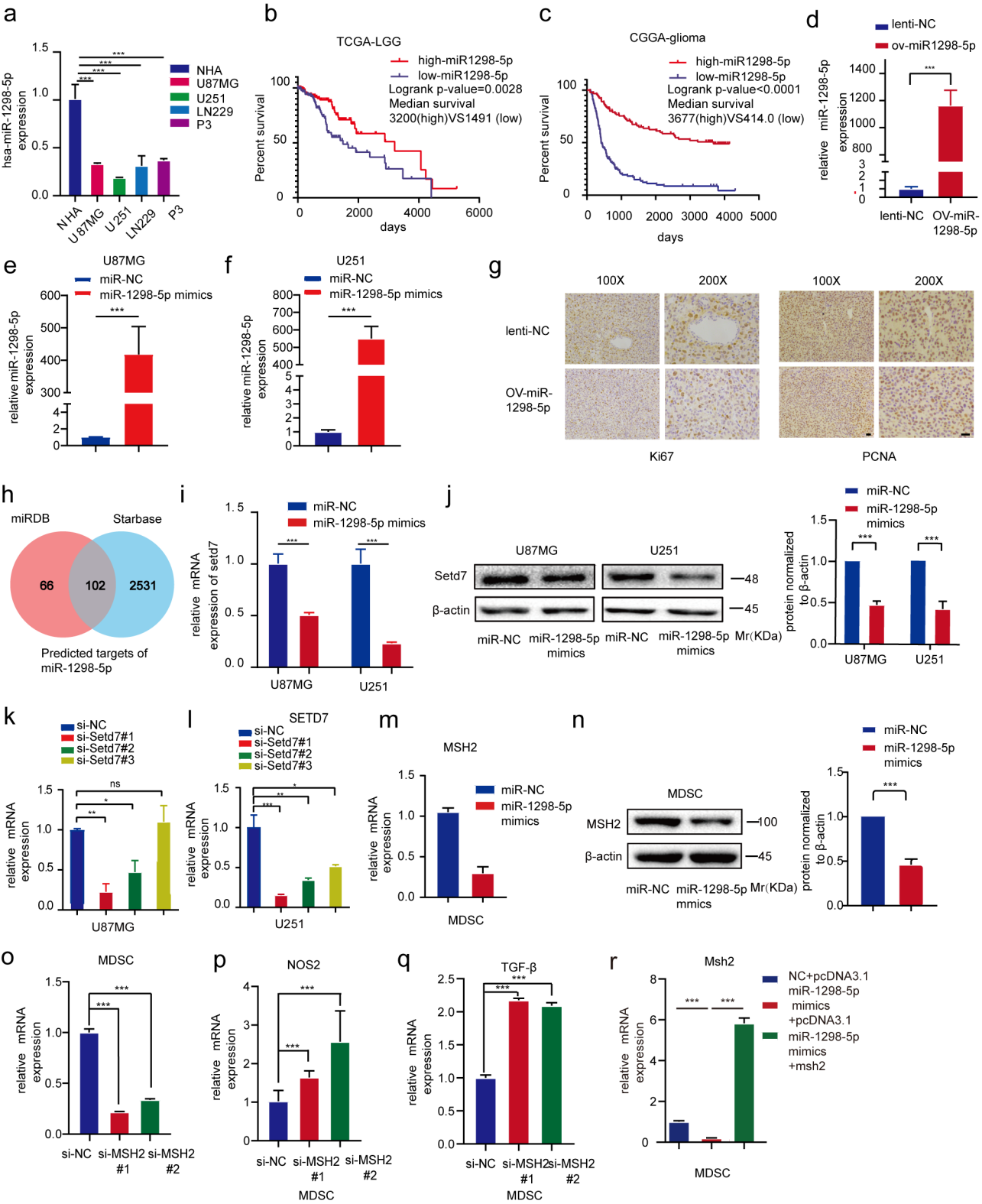
Fig. S7 miR-1298-5p could inhibit glioma proliferation and promote the immune suppressive ability of MDSCs (a) miR-1298-5p was reduced in U87MG, U251, LN229 and P3, compared with NHA. (b, c) Kaplan–Meier survival curves of patients with high and low miR-1298-5p expression in glioma, according to the TCGA and CGGA databases. P-value was obtained by log-rank t-test. (d) miR-1298-5p overexpression efficiency using lentivirus stably transfected in U87MG cells. (e, f) miR-1298-5p overexpression efficiency in U87MG, U251 cells transfected with miR-1298-5p mimics and miR-NC was examined by qRT-PCR. (g) Protein levels of Ki67 and PCNA in xenograft sections from miR-1298-5p overexpressing or negative control U87MG cell tissues were determined by IHC staining. (h) Predict the target genes of miR-1298-5p in glioma using miRDB and Starbase. (i, j) The expression of SETD7 in U87MG and U251 transfected with miR-1298-5p and miR-NC was examined by qRT-PCR and western blot. (k, l) SETD7 expression in U87MG and U251 transfected with si-NC and si-SETD7 was examined by qRT-PCR. (m, n) The expression of MSH2 in MDSCs transfected with miR-1298-5p and miR-NC was examined by qRT-PCR and western blot. (o) MSH2 expression in MDSCs transfected with si-NC and si-MSH2 was examined by qRT-PCR. (p, q) qRT-PCR demonstrated that MSH2 increased the expression of NOS2 and TGF-β in MDSCs. (r) MSH2 expression in MDSCs transfected with miR-1298-5p mimics and pcDNA3.1-Msh2 or pcDNA3.1 was examined by qRT-PCR. Data are shown as the mean ± SD of three independent experiments. Statistical significance was determined using one-way ANOVA test (*, P < 0.05; **, P < 0.01; ***, P < 0.001).


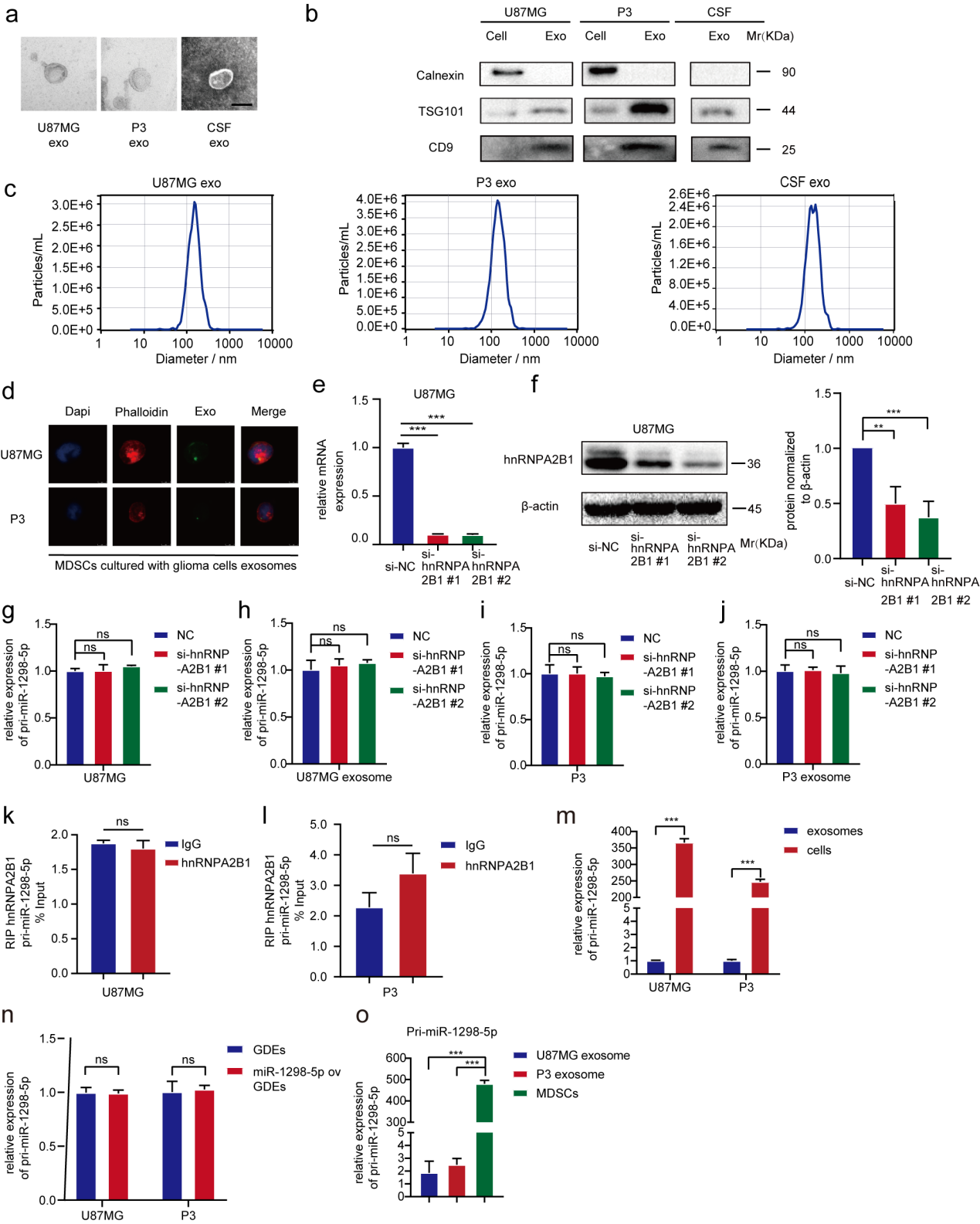


Fig. S8 Characterization of glioma exosomes and phagocytosis of glioma exosomes by MDSCs.

1. Representative transmission electron micrograph of exosomes isolated from CSF and culture supernatants of the glioma cell lines U87MG and P3. (b) Western blot analysis of the presence of CD9, TSG101 and the absence of calnexin in GDEs. (c) Exosome concentration and size distribution by qNano analysis. (d) Representative images by confocal microscopy of the internalization of PKH67-labeled glioma exosomes (green) by MDSCs. (e, f) hnRNPA2B1 expression in U87MG transfected with si-NC and si- hnRNPA2B1 was examined by qRT-PCR and western blot. (g-j) Pri-miR-1298-5p expression in cells and exosomes in U87MG and P3 transfected with si-NC and si-hnRNPA2B1 was examined by qRT-PCR. (k, l) RIP analysis using the anti-hnRNPA2B1 antibody revealed that pri-miR-1298-5p didn’t interact with hnRNPA2B1 in U87MG and P3 cells. The negative control, IgG. (m) Pri-miR-1298-5p expression in cells and exosomes in U87MG and P3 was examined by qRT-PCR. (n) Pri-miR-1298-5p expression in MDSCs treated with exosomes was examined by qRT-PCR. (o) Pri-miR-1298-5p expression in MDSCs and exosomes of U87MG and P3 was examined by qRT-PCR. Data are shown as the mean ± SD of three independent experiments. Statistical significance was determined using one-way ANOVA test (*, P < 0.05; **, P < 0.01; ***, P < 0.001).


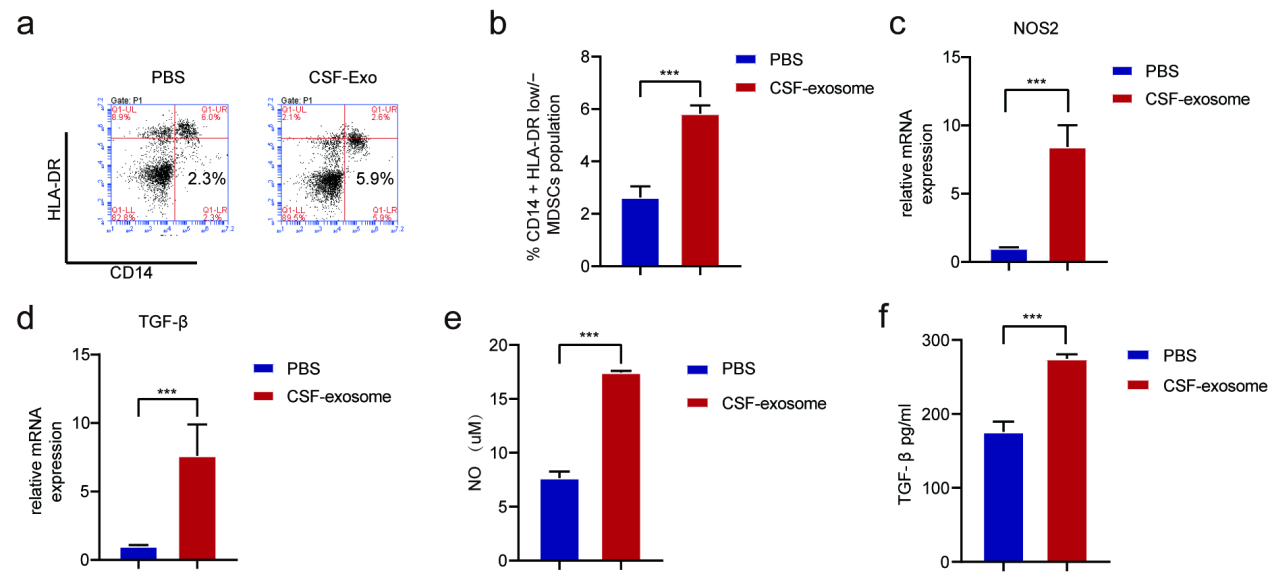


Fig. S9 Exosomes isolated from CSF promoted the immunosuppressive effects of MDSCs

(a, b) Flow cytometry assay showed that exosomes isolated from CSF upregulated the proportion of CD14+/HLA-DR low/-MDSCs population. (c, d) qRT-PCR demonstrated that exosomes isolated from CSF increased the expression of NOS2 and TGF-β in MDSCs. (e, f) NO and TGF-β in the supernatants of MDSCs were measured. Data are shown as the mean ± SD of three independent experiments. Statistical significance was determined using one-way ANOVA test (*, P < 0.05; **, P < 0.01; ***, P < 0.001).


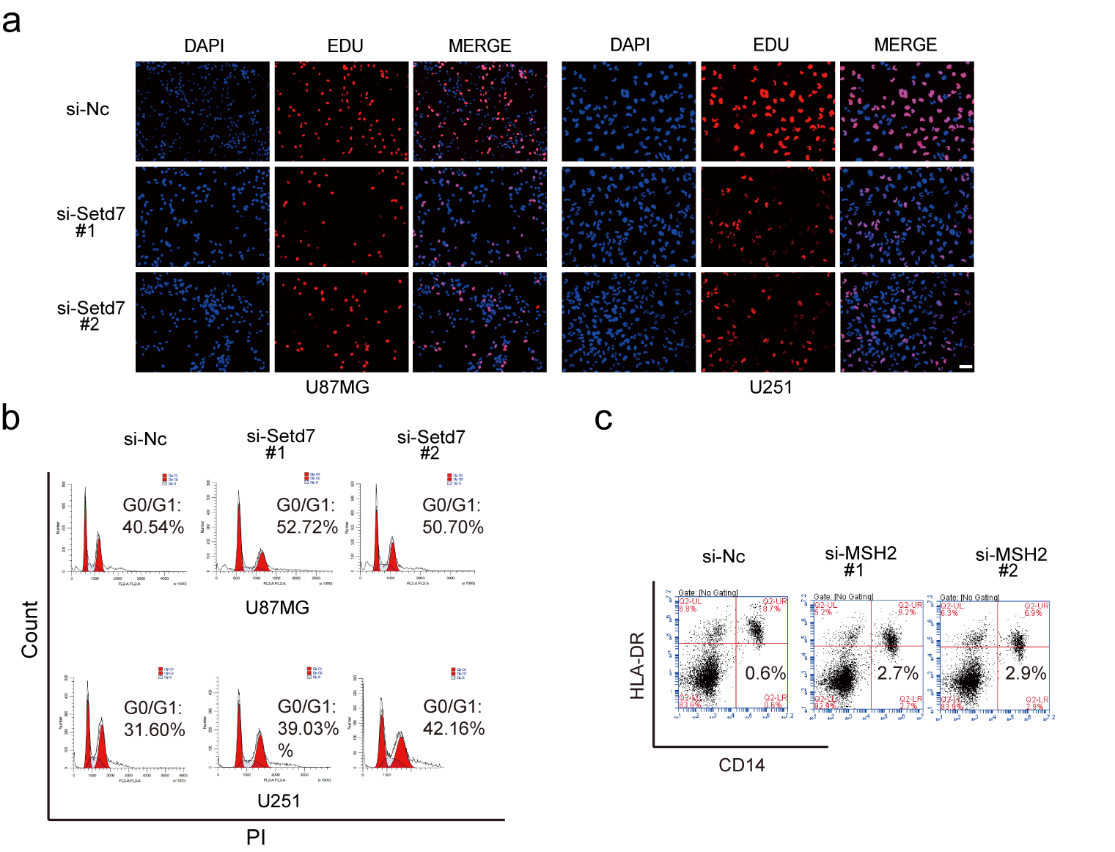


Fig. S10 miR-1298-5p targeted SETD7 in glioma and MSH2 in MDSCs

(a) The proliferation capacity of U87MG and U251 cells after SETD7 knockdown were assessed using the Edu assay. (b) Cell cycle analysis for U87MG and U251 cells knocking down SETD7. (c) Flow cytometry assay showed that MSH2 knockdown upregulated the proportion of CD14+ HLA-DRlow/− MDSCs population. Data are shown as the mean ± SD of three independent experiments. Statistical significance was determined using one-way ANOVA test (*, P < 0.05; **, P < 0.01; ***, P < 0.001).


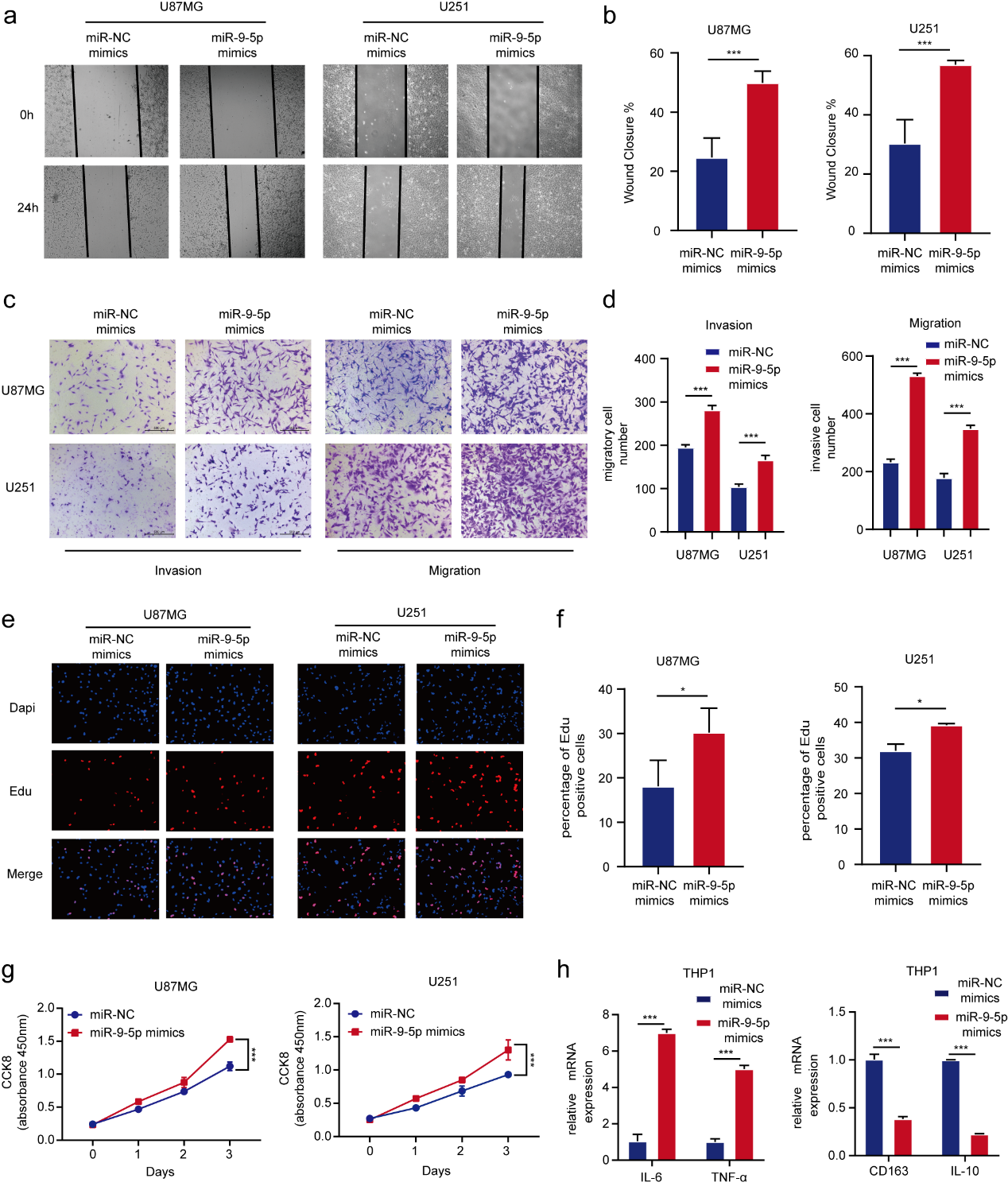
 Fig. S11 miR-9-5p could promote glioma cell proliferation, migration and invasion and induce macrophage M1 polarization

(a, b) The wound-healing assay was used to assess the migration of U87MG and U251 transfected with miR-NC and miR-9-5p mimics. Photos were taken at 0, 24h, respectively. (c, d) Non-coated transwell and Matrigel-coated transwell assays were used to determine the migration and invasion capacity of U87MG and U251 transfected with miR-NC and miR-9-5p mimics. (e-g) The proliferation ability of U87MG and U251 was determined by the Edu assay and CCK8 assay. (h) qRT-PCR demonstrated that miR-1298-5p increased the expression of IL-6 and TNF-α and reduced the level of CD163 and IL-10 in THP1. Data are shown as the mean ± SD of three independent experiments. Statistical significance was determined using one-way ANOVA test (*, P < 0.05; **, P < 0.01; ***, P < 0.001).


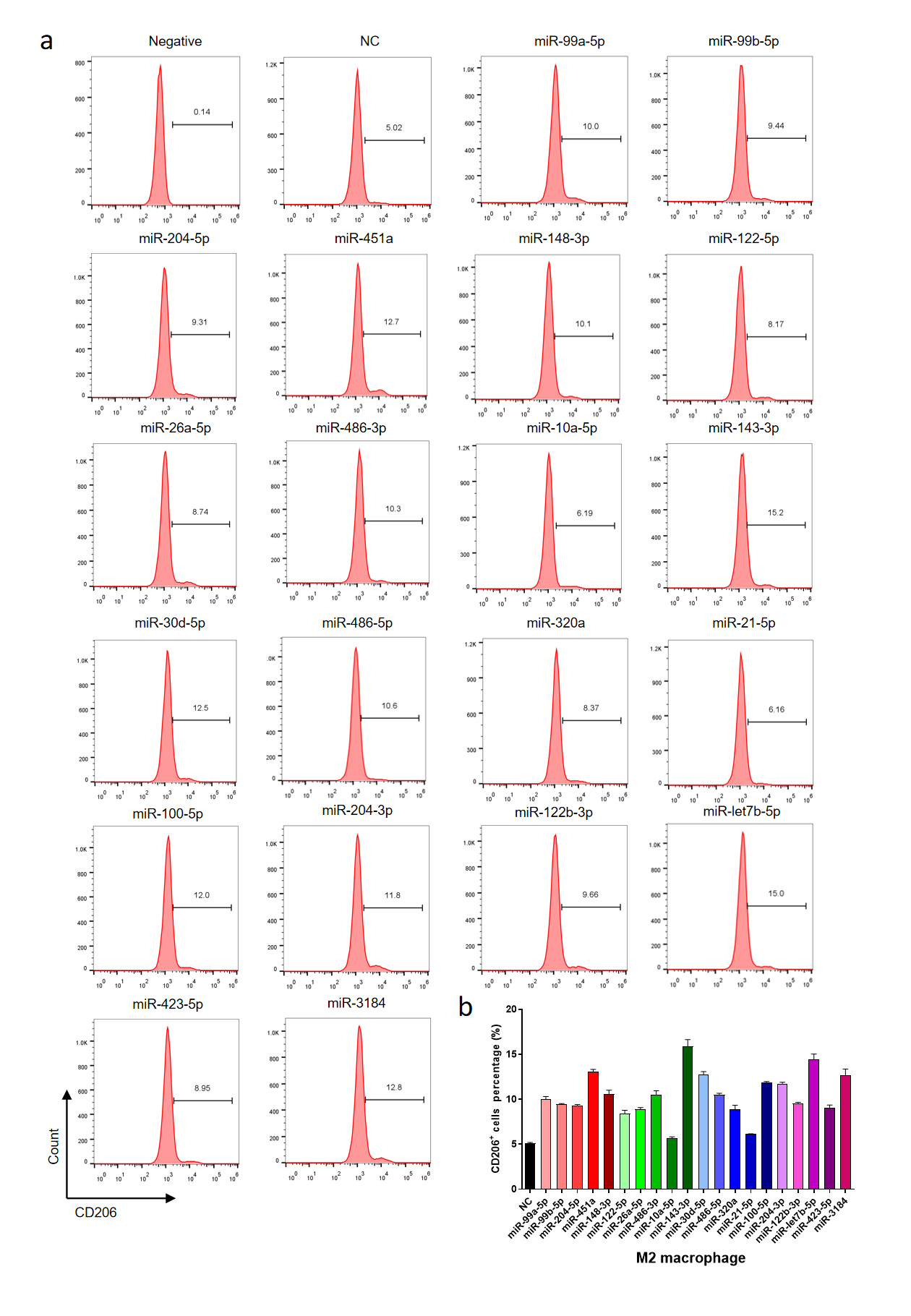


Fig. S12 CSF sEV miRNAs induced macrophage M2 polarization

(a) The flow cytometry charts of b. (b) The percentage of CD206+ macrophage in miRNA transfected macrophage. The data shown are the mean±SEM of 3 independent experiments. *P< 0.05; **P < 0.01, ***P < 0.001. Student’s t test.


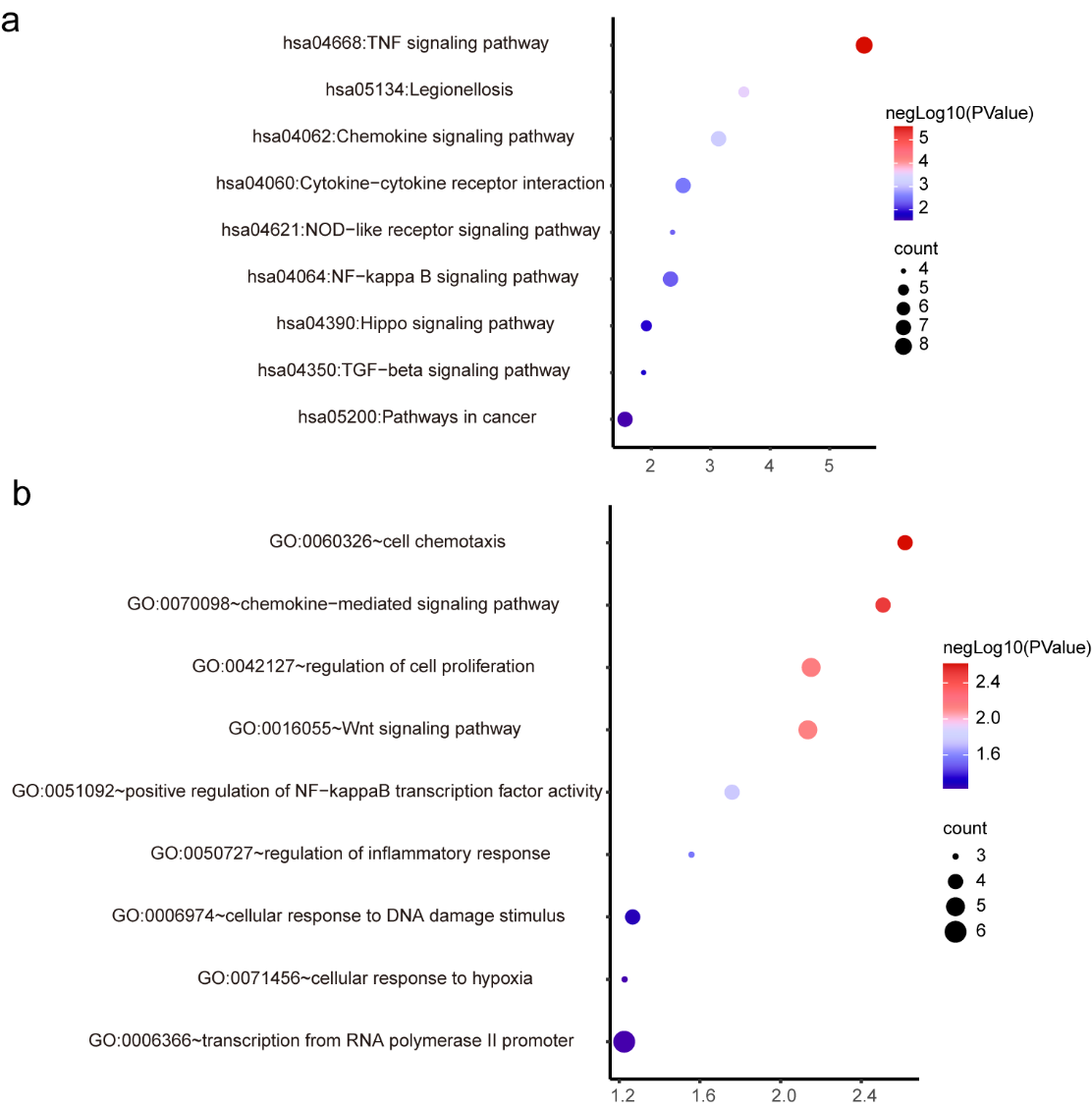


Fig. S13 GO and KEGG pathway enrichment analysis for targets of miR-1298-5p.

1. KEGG pathway enrichment analysis for targets of miR-1298-5p.(b) GO enrichment analysis for targets of miR-1298-5p


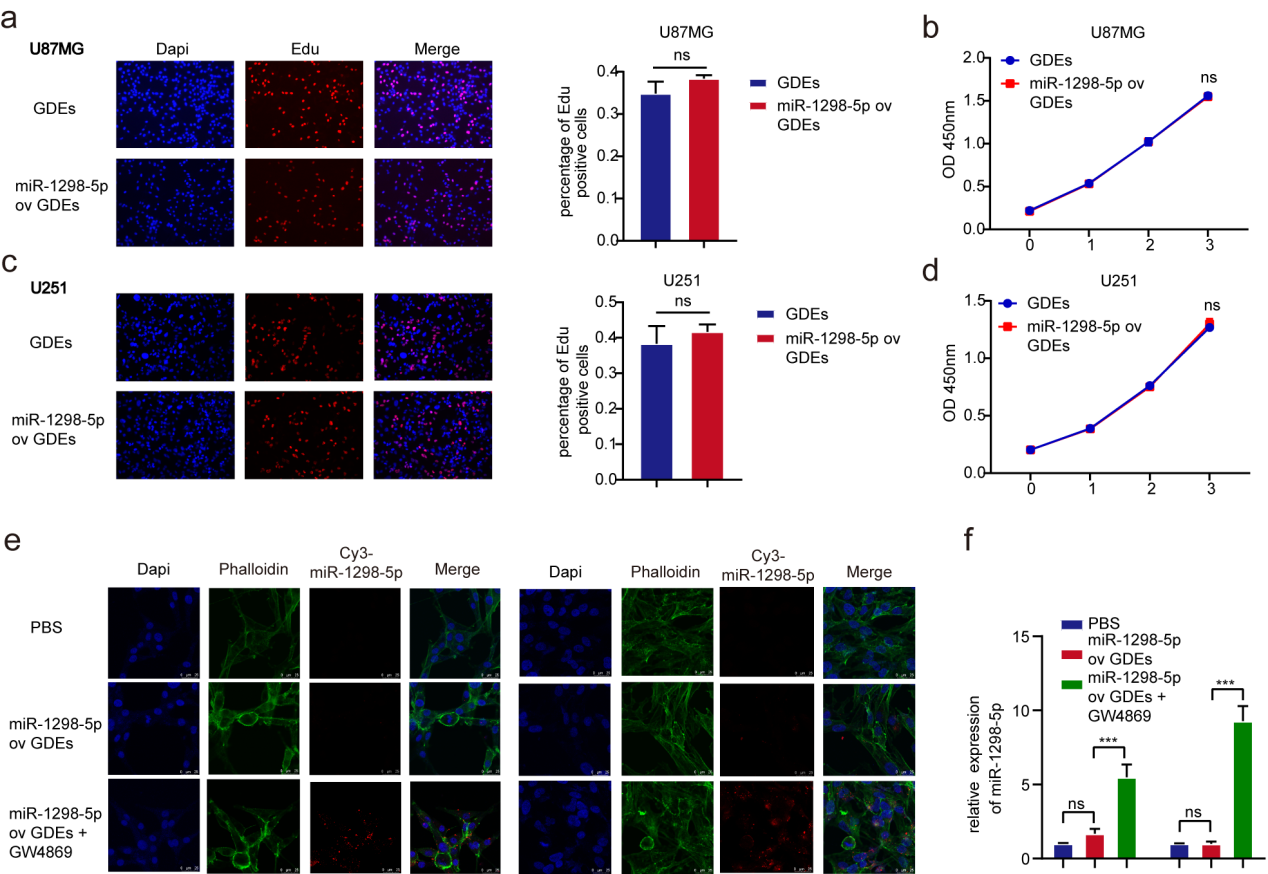


Fig. S14 miR-1298-5p-enriched exosomes didn't affect the behavior of tumor cells.

(a-d) The proliferation capacity of U87MG and U251 cells treated with GDEs or miR-1298-5p ov GDEs isolated from P3 cells were assessed using CCK8 assay and Edu assay. (e) Internalization of Cy3-labeled miR-1298-5p by U87MG and U251 cells. (f) U87MG and P3 cells were treated with miR-1298-5p ov GDEs and GW4869. qRT-PCR was performed to determine the expression of miR-1298-5p.


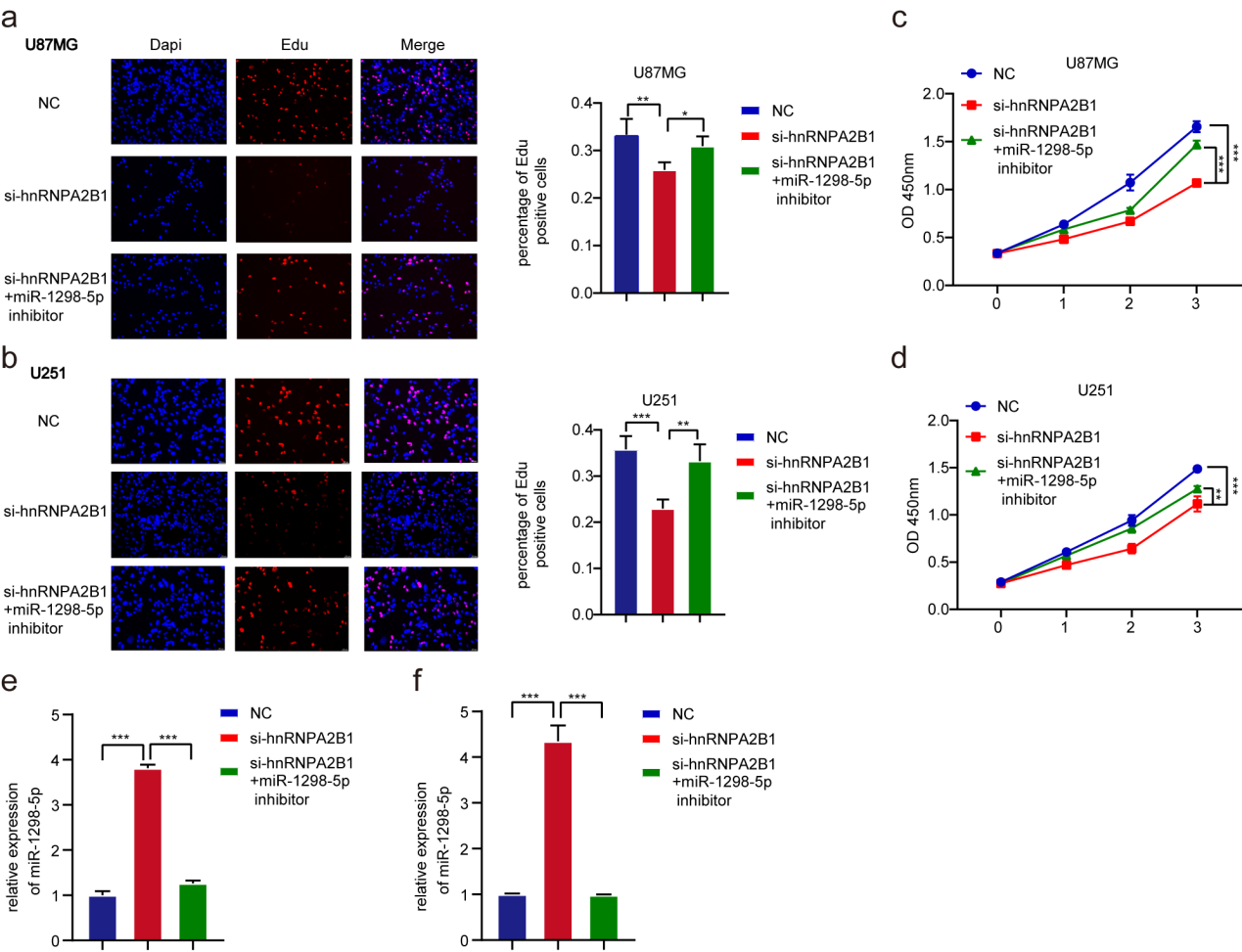


Fig. S15 The phenotypic effects of hnRNPA2B1 silencing on tumor cells. (a-d) The proliferation capacity of U87MG and U251 cells transfected with si-hnRNPA2B1 and miR-1298-5p inhibitor were assessed using CCK8 assay and Edu assay. (e-f) qRT-PCR for miR-1298-5p in of U87MG and U251 cells transfected with si-hnRNPA2B1 and miR-1298-5p inhibitor.
