# Supplementary material for: The dual role of glioma exosomal microRNAs: glioma eliminates tumor suppressor miR-1298-5p via exosomes to promote immunosuppressive effects of MDSCs
Source: Cell Death Dis. 2022 May 2;13(5):426. doi: 10.1038/s41419-022-04872-z (PMC9061735; doi:10.1038/s41419-022-04872-z)
Supplement: Supplementary file 3 — Table S1 [file 41419_2022_4872_MOESM3_ESM.docx]

**Table S1**

| Patient ID | WHO grade | Histology | Gender | Age | Positon | Tumour burden（cm^2^） | IDH mutation | TERT promoter mutation | 1p  deletion | 19q  deletion | TP53 mutation | PTEN mutation | ATRX  Mutation | BRAF V600E mutation |
| --- | --- | --- | --- | --- | --- | --- | --- | --- | --- | --- | --- | --- | --- | --- |
| 1 | Ⅳ | Glioblastoma | male | 34 | left frontal lobe | 9.53 | - | **-** | **-** | **-** | **+** | + | + | - |
| 2 | Ⅲ | - Anaplastic astrocytoma | male | 67 | left frontal lobe | 5.65 |  |  |  |  |  |  |  |  |
| 3 | Ⅱ | - [astrocytoma](javascript:;) | female | 52 | left frontal lobe | 8.88 | R132H | **-** | **-** | **-** | **+** | - | + | - |
| 4 | Ⅲ | Anaplastic astrocytoma | female | 38 | - Right frontotemporal insula | 11.02 | R132H | **-** | **-** | **-** | **+** | - | + | - |
| 5 | Ⅱ | [astrocytoma](javascript:;) | female | 48 | Right insula | 4.44 | R132H | **-** | **-** | **-** | **+** | + | + | - |
| 6 | Ⅰ | Papillary glial neuronal tumor | male | 14 | right temporal lobe | 3.03 |  |  |  |  |  |  |  |  |
| 7 | Ⅲ | Anaplastic astrocytoma | female | 33 | left frontal lobe | 13.44 | R132H | - | - | - | + | - | + | - |
| 8 | Ⅱ | [astrocytoma](javascript:;) | female | 52 | left frontal lobe | 3.46 | R132H | - | - | - | - | - | + | - |
| 9 | Ⅱ | Diffuse astrocytoma | male | 47 | right frontal lobe | 5.485 | R132H | C228T | Total deletion | Total deletion | - | - | - | - |
| 10 | Ⅳ | Glioblastoma | female | 53 | left parietal lobe | 7.84 | R172W | - | Total deletion | Total deletion | + | + | + | - |
| 11 | Ⅳ | Glioblastoma | male | 64 | right parietal lobe | 9.5 | - | C250T | Total deletion | - | + | + | - | - |
| 12 | Ⅳ | Glioblastoma | female | 70 | right frontal lobe | 9.68 |  |  |  |  |  |  |  |  |
| 13 | Ⅱ | astrocytoma | male | 51 | right frontal lobe | 10.43 | R132H | - | Total deletion | Total deletion | + | - | - | - |
| 14 | Ⅰ | astrocytoma | male | 53 | right frontal lobe | 6.17 | R132H | - | Total deletion | Total deletion | - | - | - | - |
| 15 | Ⅳ | Glioblastoma | female | 18 | Right occipital lobe | 11.68 | - | - | - | - | - | - | + | - |
| 16 | Ⅱ | Diffuse astrocytoma | male | 30 | Right frontotemporal insula | 0 |  |  |  |  |  |  |  |  |
| 17 | Ⅲ | Anaplastic astrocytoma | male | 42 | left frontal lobe | 7.8 | - | - | - | - | - | - | - | - |
| 18 | Ⅲ | - Anaplastic oligodendroglioma | male | 52 | left frontal lobe | 12.32 | R172W | - | - | Total deletion | - | - | - | - |
| 19 | Ⅱ | oligodendroglioma | male | 60 | Right occipital lobe | 11.55 | - | - | - | Total deletion | - | - | + | - |
| 20 | Ⅲ | anaplastic astrocytoma | male | 72 | left frontal lobe | 10.79 | - | - | + | - | + | - | - | - |
| 21 | Ⅱ | oligodendroglioma | male | 56 | right frontal lobe | 9.95 |  |  |  |  |  |  |  |  |
| 22 | Ⅱ | astrocytoma | female | 30 | Left Fronto parietal lobe | 10.51 | R132H | C228T | Total deletion | Total deletion | - | + | + | - |
| 23 | Ⅱ | astrocytoma | female | 50 | right temporal lobe | 6.265 |  |  |  |  |  |  |  |  |
| 24 | Ⅳ | Glioblastoma | male | 52 | right frontal lobe | 15.96 |  |  |  |  |  |  |  |  |
| 25 | Ⅱ | astrocytoma | male | 49 | left frontal lobe | 6.6 |  |  |  |  |  |  |  |  |
| 26 | Ⅳ | Glioblastoma | female | 65 | Left parietotemporal occipital lobe | 10.64 | - | C228T | - | - |  |  |  | - |
| 27 | Ⅱ | oligodendroglioma | male | 62 | right frontal lobe | 6.275 | R132H | - | - | - | - | - | - | - |
| 28 | Ⅰ | astrocytoma | male | 35 | Left cerebellar hemisphere | 3.775 | 0 | - | - | - | - | - | - | - |
| 29 | Ⅲ | Anaplastic oligodendroglioma | female | 48 | left frontal lobe | 8.96 | R132H | C228T | - | - | - | - | + | - |
| 30 | Ⅱ | Diffuse astrocytoma | female | 38 | left temporal lobe | 7.91 | R132H | - | - | - | + | - | + | - |
| 31 | Ⅲ | oligodendroglioma | male | 55 | Left frontotemporal lobe | 6.02 | R132H | C228T | Total deletion | Total deletion | - | + | - | - |
| 32 | Ⅳ | Glioblastoma | female | 60 | Left frontal parietal temporal lobe | 9.82 | - |  | + | - | - | + | - | - |
| 33 | Ⅳ | Glioblastoma | male | 45 | left frontal lobe | 10.81 | R132H | - | - | - | + | - | + | - |
| 34 | Ⅳ | Glioblastoma | male | 61 | left frontal lobe | 8.77 | - | - | - | - | - | - | - | - |
| 35 | Ⅲ | anaplastic astrocytoma | male | 41 | Left insula | 8.54 |  |  |  |  |  |  |  |  |
| 36 | Ⅱ | Central neurocytoma | female | 27 | Left lateral ventricle | 10.71 |  |  |  |  |  |  |  |  |
| 37 | Ⅰ | Ganglioglioma | female | 32 | Left lateral ventricle | 2.86 |  |  |  |  |  |  |  |  |
| 38 | Ⅳ | Glioblastoma | female | 66 | Right occipital lobe | 8.4 | - | - | - | Total deletion | - | - | + | - |
| 40 | Ⅲ | astrocytoma | male | 39 | right frontal lobe | 5.2 |  |  |  |  |  |  |  |  |
| 41 | Ⅱ | Diffuse astrocytoma | female | 63 | Left frontal parietal lobe | 6.8 |  |  |  |  |  |  |  |  |
| 42 | Ⅱ | oligodendroglioma | female | 49 | right frontal lobe | 9.56 | R132H | - | Total deletion | Total deletion | + | - | + | - |
| 43 | Ⅳ | Glioblastoma | female | 55 | left frontal lobe | 11.67 |  |  |  |  |  |  |  |  |
| 44 | Ⅳ | Glioblastoma | male | 62 | left parietal lobe | 9.67 | - | - | - | - | - | + | + | - |
| 45 | Ⅱ | Pleomorphic astrocytoma | Male | 19 | left frontal lobe | 5.17 |  |  |  |  |  |  |  |  |

|  |  |  |  |  |
| --- | --- | --- | --- | --- |
