# Supplementary material for: The dual role of glioma exosomal microRNAs: glioma eliminates tumor suppressor miR-1298-5p via exosomes to promote immunosuppressive effects of MDSCs
Source: Cell Death Dis. 2022 May 2;13(5):426. doi: 10.1038/s41419-022-04872-z (PMC9061735; doi:10.1038/s41419-022-04872-z)
Supplement: Supplementary file 4 — Table S2 [file 41419_2022_4872_MOESM4_ESM.docx]

Table S2 The tumour burden (valuated by SPD) and distance between the tumour and CSF circulation pathway (CSF type)

| Patient ID | CSF type | SPD | Mean_Readcounts_adj |
| --- | --- | --- | --- |
| 7pre | YES | 13.44 | 203.1061 |
| 33pre | YES | 10.81 | 196.1391 |
| 13pre | YES | 10.43 | 136.4224 |
| 23pre | NO | 6.265 | 125.9714 |
| 37pre | YES | 2.86 | 116.5807 |
| 41pre | NO | 6.8 | 113.0726 |
| 12pre | NO | 9.68 | 105.3034 |
| 26pre | YES | 10.64 | 103.5834 |
| 18pre | YES | 12.32 | 97.26484 |
| 8pre | NO | 3.46 | 96.97364 |
| 42pre | NO | 9.56 | 94.55521 |
| 38pre | YES | 8.4 | 94.22561 |
| 9pre | YES | 5.485 | 91.74305 |
| 21pre | NO | 9.95 | 91.68161 |
| 31pre | YES | 6.02 | 91.58717 |
| 3pre | YES | 8.88 | 88.92154 |
| 15pre | YES | 11.68 | 85.8599 |
| 4pre | YES | 11.02 | 82.3626 |
| 30pre | NO | 7.91 | 81.1428 |
| 2pre | YES | 5.65 | 76.30199 |
| 6pre | NO | 3.03 | 75.01183 |
| 28pre | NO | 3.775 | 72.41137 |
| 44pre | YES | 9.67 | 70.48174 |
| 16pre | NO | 13.04 | 61.10357 |
| 11pre | YES | 9.5 | 58.08551 |
| 10pre | YES | 7.84 | 57.72935 |
| 29pre | NO | 8.96 | 57.50042 |
| 1pre | YES | 9.53 | 55.19863 |
| 25pre | YES | 6.6 | 54.2966 |
| 19pre | NO | 11.55 | 50.71025 |
| 35pre | NO | 8.54 | 50.23142 |
| 22pre | YES | 10.51 | 47.23516 |
| 24pre | YES | 15.96 | 44.55915 |
| 40pre | NO | 5.2 | 39.03508 |
| 32pre | NO | 9.82 | 37.88667 |
| 5pre | NO | 4.44 | 31.34932 |
| 14pre | NO | 6.17 | 29.95081 |

wilcoxon-test indicates miRNA abundance in CSF exosomes of glioma patients was not different between CSF types (p=0.18). Pearson Correlation analysis indicates miRNA abundance in CSF exosomes of glioma patients was not associated with position (r=0.14 and p=0.39)
