# Supplementary material for: The dual role of glioma exosomal microRNAs: glioma eliminates tumor suppressor miR-1298-5p via exosomes to promote immunosuppressive effects of MDSCs
Source: Cell Death Dis. 2022 May 2;13(5):426. doi: 10.1038/s41419-022-04872-z (PMC9061735; doi:10.1038/s41419-022-04872-z)
Supplement: Supplementary file 6 — Table S4 [file 41419_2022_4872_MOESM6_ESM.docx]

| Table S4 The functional annotation of high-expressed CSF exosomal miRNAs | | | | | | |
| --- | --- | --- | --- | --- | --- | --- |
| Up/down  in CSF | miRNA types | miRNAs | *P* adj-CSF | Downstream  target gene | Phenotype | PMID |
| Up-regulated in CSF exosome | TS-miR in glioma | hsa-let-7b-5p | 1.09E-06 | PLK1, CCNA2, cyclin B2 | migration, invasion | 30995921 |
|  |  | hsa-let-7c-5p | 2.71E-19 | E2F5 | Proliferation, Invasion, and Migration | 29362021 |
|  |  | hsa-miR-100-5p | 8.28E-13 | SMRT NCOR2 | proliferation | 24244722 |
|  |  | hsa-miR-105-5p | 4.85E-15 | SUZ12 | proliferation, tumorigenesis, migration, invasion, and drug sensitivity | 28618952 |
|  |  | hsa-miR-124-3p | 6.37E-05 | Fra-2 | aggressiveness | 30243808 |
|  |  | hsa-miR-125a-5p | 7.46E-34 | TAZ | proliferation | 25542152 |
|  |  | hsa-miR-125b-5p | 3.84E-25 |  | proliferation migration | 30131528 |
|  |  | hsa-miR-128-3p | 8.67E-21 | NPTX1 | proliferation | 30906495 |
|  |  | hsa-miR-129-5p | 6.34E-05 | Wnt5a | proliferation migration and angiogenesis | 29531296 |
|  |  | hsa-miR-1298-5p | 4.86E-05 | SETD 7 | cell proliferation and apoptosis | 28762861 |
|  |  | hsa-miR-139-5p | 5.69E-08 | ELTD1 | suppressed cell proliferation and inducing apoptosis，regulating cell cycle | 26449464 |
|  |  | hsa-miR-145-3p | 1.82E-08 | WISP1 | inhibited cell proliferation, invasion, migration | 31800303 |
|  |  | hsa-miR-151a-3p | 0.000191 | XRCC4 | TMZ resistance to chemoresponsive | 30992025 |
|  |  | hsa-miR-193a-5p | 1.01E-05 | NOVA1 | proliferation, migraton | 30304561 |
|  |  | hsa-miR-195-3p | 6.61E-21 | Caspase-3, -8, -9 and Bcl-2 | cycle arrest | 30806889 |
|  |  | hsa-miR-200c-3p | 0.0002 | HMGB3 | proliferation metastatic | 30232806 |
|  |  | hsa-miR-204-3p | 7.23E-12 | IGFBP2 | apoptosis | 27487563 |
|  |  | hsa-miR-204-5p | 5.86E-34 | RAB22A | Invasive | 26134825 |
|  |  | hsa-miR-224-5p | 4.82E-10 | mTOR | migration Invasive | 31179240 |
|  |  | hsa-miR-370-3p | 2.05E-05 | β-catenin | proliferation | 27138069 |
|  |  | hsa-miR-382-5p | 3.79E-07 | YBX1 | proliferation, Invasive migration | 31417288 |
|  |  | hsa-miR-409-3p | 1.19E-21 | HMGN5 | proliferation, Invasive | 28109076 |
|  |  | hsa-miR-433-3p | 1.36E-06 | CREB | proliferation, Resistance to chemotherapy | 27926502 |
|  |  | hsa-miR-483-5p | 4.06E-11 | ERK1 | proliferation | 22465663 |
|  |  | hsa-miR-504-5p | 6.84E-22 | FZD7 | EMT | 31419987 |
|  |  | hsa-miR-543 | 5.19E-20 |  | proliferation, Invasive migration | 28627653 |
|  |  | hsa-miR-708-3p | 0.000912 | ZEB1 | proliferation, Invasive | 26556542、29575368 |
|  |  | hsa-miR-99a-5p | 2.28E-27 | FGFR3 and PI3K/Akt | proliferation, Invasive | 23409016、23298836 |
|  |  | hsa-miR-99b-5p | 5.63E-37 |  | proliferation, Invasive | 27382299 |
|  | TS-miR in other tumours | hsa-let-7e-5p | 6.90E-14 | Chemokine Receptor 7 | Proliferation and Metastasis | 31205553 |
|  |  | hsa-miR-125b-1-3p | 1.68E-07 | S1PR1 | invasion | 30082521 |
|  |  | hsa-miR-1298-3p | 1.81E-05 | BRD9 | proliferation invasion | 30660651 |
|  |  | hsa-miR-30a-3p | 4.17E-15 | DNMT3A | proliferation cycle arrest | 31695416 |
|  |  | hsa-miR-30c-2-3p | 0.001413 | RAB31 | proliferation apoptosis | 30534536 |
|  |  | hsa-miR-30c-5p | 1.28E-25 | SOX9 | proliferation Invasive migration apoptosis | 31541993 |
|  |  | hsa-miR-323a-3p | 7.83E-08 | MET/SMAD3/SNAIL | EMT | 28837140 |
|  |  | hsa-miR-328-3p | 2.04E-12 | HDGF HIF1AN | early development | 31148183 |
|  |  | hsa-miR-335-3p | 3.90E-19 | ABCA3 | Resistance to chemotherapy | 30639603 |
|  |  | hsa-miR-340-3p | 7.87E-08 | CIKIL-2 | proliferation | 25826780 |
|  |  | hsa-miR-34b-3p | 3.05E-11 | CDK4 | proliferation | 31199581 |
|  |  | hsa-miR-375-3p | 7.79E-07 | YAP1、SP1 | proliferation | 31543507 |
|  |  | hsa-miR-455-5p | 0.000357 | IGF-1R | proliferation, Invasive | 31732382 |
|  |  | hsa-miR-654-3p | 1.14E-07 |  | proliferation | 28030816 |
|  |  | hsa-miR-99b-3p | 2.32E-09 | glycogen synthase kinase-3β |  | 26315788 |
|  | TP-miR | hsa-miR-10a-5p | 9.87E-33 | PTEN | progression | 30927504 |
|  |  | hsa-miR-10b-5p | 1.70E-19 | Apaf-1 | proliferation | 29514931 |
|  |  | hsa-miR-127-3p | 0.00021 | 7-Sep | migration and invasion | 24604520 |
|  |  | hsa-miR-149-5p | 2.15E-10 | caspase-2 | cell viability, inhibition of apoptosis | 27049919 |
|  |  | hsa-miR-196a-5p | 9.34E-09 | FOXO1 | proliferation Invasive migration | 28666797 |
|  |  | hsa-miR-222-3p | 3.58E-17 | p27 | proliferation Invasive migration | 31105847 |
|  |  | hsa-miR-92b-3p | 1.28E-25 | Homeobox D10 | proliferation, Invasive | 31106881 |
|  |  | hsa-miR-92b-5p | 4.85E-15 | NLK | proliferation, Invasive | 23416699 |
|  | Not known | hsa-let-7d-3p | 3.40E-07 | Not known | Not known | Not known |
|  |  | hsa-miR-1180-3p | 1.25E-13 |  |  |  |
|  |  | hsa-miR-125b-2-3p | 2.93E-07 |  |  |  |
|  |  | hsa-miR-1911-5p | 4.06E-11 |  |  |  |

| **TableS4 The** **functional annotation of high-expressed CSF exosomal miRNAs (continued)** | | | | | | |
| --- | --- | --- | --- | --- | --- | --- |
| **Up/down**  **in CSF** | **miRNA types** | **miRNAs** | ***P* adj-CSF** | **Downstream**  **target gene** | **Phenotype** | **PMID** |
| **Down-**  **regulated in CSF exosome** | **TS-miR in glioma** | hsa-let-7g-5p | 3.07E-32 | VSIG4 | EMT | 27634309 |
|  |  | hsa-let-7i-5p | 9.89E-19 | IKBKE | invasion and migration | 25656572 |
|  |  | hsa-miR-101-3p | 6.44E-26 | TRIM44 | EMT | 30539341 |
|  |  | hsa-miR-103a-3p | 3.10E-08 | FEZF1 CDC25A | proliferation migration and invasion | 28651608 |
|  |  | hsa-miR-103b | 2.41E-08 | SALL4 | Proliferation and Migration | 30423818 |
|  |  | hsa-miR-107 | 1.51E-08 | notch2 | growth invasion | 23572380 |
|  |  | hsa-miR-140-5p | 3.28E-07 | ADAM9 | inhibited proliferation, migration and invasion | 27498787 |
|  |  | hsa-miR-142-3p | 4.43E-22 | ITGB8 | inhibited cell proliferation and invasion | 29337055 |
|  |  | hsa-miR-142-5p | 6.60E-34 | Rac1 | inhibits the migration and invasion | 28714015 |
|  |  | hsa-miR-146a-5p | 8.75E-41 | SMAD4 | suppresses tumorigenic gene, MMP9 in gliomaassociated microglia and glioma cell viability | 29861845 |
|  |  | hsa-miR-146b-5p | 1.90E-25 | MMP16 | migration and invasion | 31385537 |
|  |  | hsa-miR-148b-3p | 1.37E-14 | HOTAIR | cell proliferation, cell cycle progression and invasion | 27446363 |
|  |  | hsa-miR-150-5p | 1.98E-14 | PLP2 | proliferation, migration and invasion, and resistance to temozolomide | 31535380 |
|  |  | hsa-miR-155-5p | 5.27E-12 | FAM133A | Invasion and migration | 29885519 |
|  |  | hsa-miR-15b-5p | 0.01001 | SALL4 | proliferation, migration | 30423818 |
|  |  | hsa-miR-16-5p | 4.53E-19 | URGCP | growth invasion | 29044221 |
|  |  | hsa-miR-185-5p | 9.84E-09 | HGMA2 | proliferation，metastatic | 30860979 |
|  |  | hsa-miR-186-5p | 2.80E-28 | FGF2 REIA | invasion migration | 28213656 |
|  |  | hsa-miR-199b-5p | 0.005377 | HES1 | proliferation | 26823491 |
|  |  | hsa-miR-21-5p | 3.28E-28 | p-21 protein | proliferation | 25646699 |
|  |  | hsa-miR-223-5p | 1.10E-11 |  | Resistance to chemotherapy | 31046428 |
|  |  | hsa-miR-29a-3p | 2.88E-09 | Notch2 | proliferation | 29258209 |
|  |  | hsa-miR-30e-5p | 2.95E-08 | MYBL2 | Invasive migration cycle arrest | 30305611 |
|  |  | hsa-miR-340-5p | 4.85E-15 | POSTN | proliferation TAM | 31427735 |
|  |  | hsa-miR-342-3p | 6.56E-06 | E2F1 | proliferation | 30868897 |
|  |  | hsa-miR-378c | 9.03E-29 | IRG1 | Invasive migration | 29949160 |
|  |  | hsa-miR-542-3p | 0.03136 | AKT | Invasive | 26286747 |
|  |  | hsa-miR-7-5p | 1.42E-05 | PI3K and Raf-1 | proliferation | 24603851 |
|  |  | hsa-miR-93-5p | 1.75E-10 | MMP2 | proliferation, Invasive | 31773703 |
|  | **TS-miR in other tumours** | hsa-miR-140-3p | 4.92E-25 | BRD9 | proliferation | 30660651 |
|  |  | hsa-miR-16-2-3p | 0.000215 | FGFR2 | enhances apoptosis and chemosensitivity | 31018244 |
|  |  | hsa-miR-181b-5p | 3.11E-05 | NOVA1 | proliferation, migraton | 25299073 |
|  |  | hsa-miR-23a-3p | 1.23E-13 | ADCY1 | proliferation migration Invasive | 30867808 |
|  |  | hsa-miR-26b-5p | 1.51E-16 | TRIM44 | Invasive migration apoptosis proliferation | 30922374 |
|  |  | hsa-miR-27a-5p | 2.11E-16 | EGFR | proliferation | 29415999 |
|  |  | hsa-miR-378a-3p | 1.59E-22 |  | proliferation | 29897167 |
|  |  | hsa-miR-451a | 3.67E-06 | ATF2 | Invasive migration | 30229828 |
|  |  | hsa-miR-660-5p | 3.55E-06 | MDM2 | proliferation | 25501825 |
|  | **TP-miR** | hsa-miR-106b-5p | 1.89E-08 | RBL1 RBL2 CASP8 | proliferation | 24166509 |
|  |  | hsa-miR-17-5p | 2.64E-08 |  |  | 24145352 |
|  |  | hsa-miR-20a-5p | 3.97E-08 | TIMP2 | proliferation Invasive migration | 24704830 |
|  |  | hsa-miR-215-5p | 0.01258 | PCDH9 | proliferation metastatic apoptosis | 28055966 |
|  |  | hsa-miR-23b-3p | 3.76E-05 | ATG12 | apoptosis | 29162158 |
|  |  | hsa-miR-27a-3p | 1.86E-07 | MXI1 | proliferation | 23254855 |
|  |  | hsa-miR-29b-3p | 1.77E-07 | CNR1 | chemotherapy | 27613640 |
|  |  | hsa-miR-330-5p | 0.000786 | SH3GL2 | proliferation | 23029364 |
|  |  | hsa-miR-374a-5p | 1.89E-08 | FOXO1 | Resistance to chemotherapy | 30841958 |
|  |  | hsa-miR-374b-5p | 2.64E-15 | GATA3 | proliferation, Invasive | 30868892 |
|  |  | hsa-miR-454-3p | 0.005873 | EGR3. | proliferation | 31641382 |
|  |  | hsa-miR-500a-3p | 2.28E-08 | STAT3 | Stem cell resistance | 28750679 |
|  |  | hsa-miR-582-3p | 0.005969 | AXIN2, DKK3 and SFRP1 | tumorigenesis and tumour recurrence | 26468775 |
|  |  | hsa-miR-629-5p | 1.98E-14 | β-catenin | proliferation, migration | 30954576 |
|  |  | hsa-miR-652-3p | 3.08E-22 | KCNN3 | proliferation | 31696467 |
|  |  | hsa-miR-769-5p | 5.13E-05 | Lysine Methyltransferase 2A | proliferation | 31807002 |
|  | **Not known** | hsa-miR-106b-3p | 1.65E-13 | Not known | Not known | Not known |
|  |  | hsa-miR-181a-3p | 6.16E-05 |  |  |  |
|  |  | hsa-miR-3529-3p | 1.42E-05 |  |  |  |
|  |  | hsa-miR-374a-3p | 5.99E-06 |  |  |  |
|  |  | hsa-miR-374c-3p | 2.64E-15 |  |  |  |
