# Supplementary material for: The dual role of glioma exosomal microRNAs: glioma eliminates tumor suppressor miR-1298-5p via exosomes to promote immunosuppressive effects of MDSCs
Source: Cell Death Dis. 2022 May 2;13(5):426. doi: 10.1038/s41419-022-04872-z (PMC9061735; doi:10.1038/s41419-022-04872-z)
Supplement: Supplementary file 8 — Table S6 [file 41419_2022_4872_MOESM8_ESM.docx]

**Table S6.** Sequences for siRNA, microRNA mimics and primers for qRT-PCR

| Sequences for siRNA, microRNA mimics and primers for qRT-PCR |
| --- |
| GAPDH forward: 5′-GCACCGTCAAGGCTGAGAAC-3′ |
| GAPDH reverse: 5′-TGGTGAAGACGCCAGTGGA-3′ |
| CD163 forward: 5′-GGCTTGCAGTTTCCTCAAGA-3′ |
| CD163 reverse: 5′-GACACAGAAATTAGTTCAGCAGCA-3′ |
| SETD7 forward: 5′-ATGGATAGCGACGACGAGATG-3′ |
| SETD7 reverse: 5′-GCAGAACCCGTGCGGTAAT-3′ |
| NOS2 forward: 5’-CGTCCTGTCCCCTTTCTACTA-3′ |
| NOS2 reverse: 5’-GCCTCTGATTTTCCTGTCTCT-3′ |
| TGFB1 forward: 5′-CTGCAAGTGGACATCAACGG-3′ |
| TGFB1 reverse: 5′-TCCGTGGAGCTGAAGCAATA-3′ |
| MSH2 forward: 5′-TGGATCAGGTGGAAAACCAT-3′ |
| MSH2 reverse: 5′-ATCCAAACTGTGCACTGGAA-3′ |
| Pri-mir-1298-5p forward: 5′-GAGGAGTTAAGAGTTCATTCGGC-3′ |
| Pri-mir-1298-5p reverse: 5′-TCAGTTGCCCAGATGTATTTATTGC-3′ |
| miR-1298-5p forward: 5′-ACACTCCAGCTGGGTTCATTCGGCTGTCCA-3′ |
| miR-1298-5p reverse: 5′-TGGTGTCGTGGAGTCG-3′ |
| U6 forward: 5′-ATTGGAACGATACAGAGAAGATT-3′ |
| U6 reverse: 5′-GGAACGCTTCACGAATTTG-3′ |
| hnRNPA2B1 siRNA: 5′-AGACAAGAAAUGCAGGAAGUC-3′  3′-GACUUCCUGCAUUUCUUGUCU-5′ |
| hsa-miR-99a-5p mimics: 5′-AACCCGUAGAUCCGAUCUUGUG-3′  3′-CAAGAUCGGAUCUACGGGUUUU-5′ |
| hsa-miR-99b-5p mimics: 5′-CACCCGUAGAACCGACCUUGCG-3′  3′-CAAGGUCGGUUCUACGGGUGUU-5′ |
| hsa-miR-100-5p mimics: 5′- AACCCGUAGAUCCGAACUUGUG-3′  3′- CAAGUUCGGAUCUACGGGUUUU-5′ |
| hsa-miR-204-5p mimics: 5′- UUCCCUUUGUCAUCCUAUGCCU-3′  3′- GCAUAGGAUGACAAAGGGAAUU-5′ |
| hsa-miR-204-3p mimics: 5′- GCUGGGAAGGCAAAGGGACGU-3′  3′- GUCCCUUUGCCUUCCCAGCUU-5′ |
| hsa-miR-451a mimics: 5′- AAACCGUUACCAUUACUGAGUU-3′  3′- CUCAGUAAUGGUAACGGUUUUU-5′ |
| hsa-miR-148a-3p mimics: 5′- UCAGUGCACUACAGAACUUUGU-3′  3′- AAAGUUCUGUAGUGCACUGAUU-5′ |
| hsa-miR-122-5p mimics: 5′- UGGAGUGUGACAAUGGUGUUUG-3′  3′- AACACCAUUGUCACACUCCAUU-5′ |
| hsa-miR-122-3p mimics: 5′-AAACACCAUUGUCACACUCCAC -3′  3′- GGAGUGUGACAAUGGUGUUUUU-5′ |
| hsa-miR-let7b mimics: 5′-UGAGGUAGUAGGUUGUGUGGUU -3′  3′- CCACACAACCUACUACCUCAUU-5′ |
| hsa-miR-26a-5p mimics: 5′-UUCAAGUAAUCCAGGAUAGGCU-3′  3′- CCUAUCCUGGAUUACUUGAAUU-5′ |
| hsa-miR-9-5p mimics: 5′-UCUUUGGUUAUCUAGCUGUAUGA-3′  3′-AUACAGCUAGAUAACCAAAGAUU-5′ |
| hsa-miR-10a-5p mimics: 5′-UACCCUGUAGAUCCGAAUUUGUG-3′  3′-CAAAUUCGGAUCUACAGGGUAUU-5′ |
| hsa-miR-143-3p mimics: 5′-UGAGAUGAAGCACUGUAGCUC-3′  3′-GCUACAGUGCUUCAUCUCAUU-5′ |
| hsa-miR-423-5p mimics: 5′-UGAGGGGCAGAGAGCGAGACUUU-3′  3′-AGUCUCGCUCUCUGCCCCUCAUU-5′ |
| hsa-miR-3184-3p mimics: 5′-AAAGUCUCGCUCUCUGCCCCUCA-3′  3′-AGGGGCAGAGAGCGAGACUUUUU-5′ |
| hsa-miR-30d-5p mimics: 5′-UGUAAACAUCCCCGACUGGAAG-3′  3′-UCCAGUCGGGGAUGUUUACAUU-5′ |
| hsa-miR-486-5p mimics: 5′-UCCUGUACUGAGCUGCCCCGAG-3′  3′-CGGGGCAGCUCAGUACAGGAUU-5′ |
| hsa-miR-486-3p mimics: 5′-CGGGGCAGCUCAGUACAGGAU-3′  3′-CCUGUACUGAGCUGCCCCGUU-5′ |
| hsa-miR-320a-3p mimics: 5′-AAAAGCUGGGUUGAGAGGGCGA-3′  3′-GCCCUCUCAACCCAGCUUUUUU-5′ |
| hsa-miR-1298-5p mimics: 5′-UUCAUUCGGCUGUCCAGAUGUA-3′  3′-CAUCUGGACAGCCGAAUGAAUU-5′ |
