# Supplementary figures and images for: The dual role of glioma exosomal microRNAs: glioma eliminates tumor suppressor miR-1298-5p via exosomes to promote immunosuppressive effects of MDSCs
Source: Cell Death Dis. 2022 May 2;13(5):426. doi: 10.1038/s41419-022-04872-z (PMC9061735; doi:10.1038/s41419-022-04872-z)

Figure2c

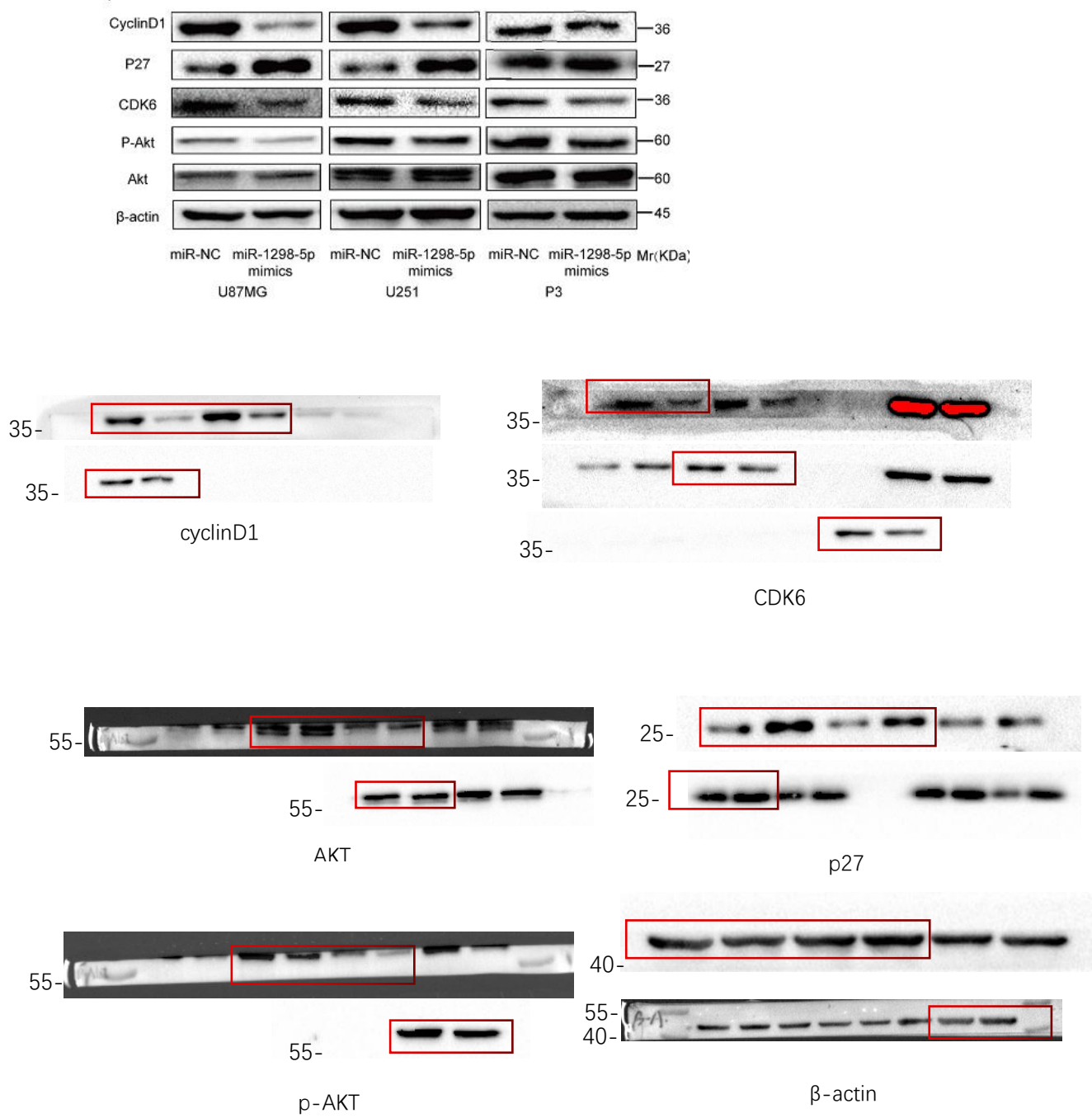

Figure3o

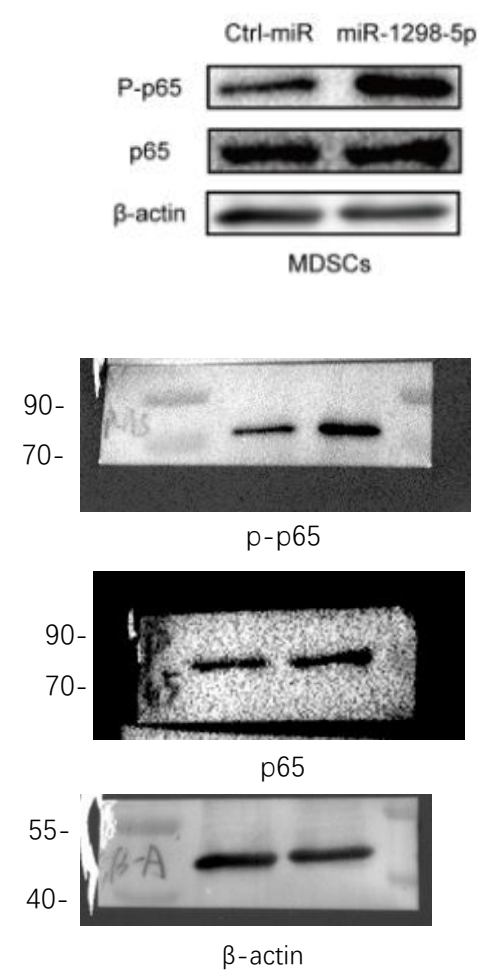

Figure4d

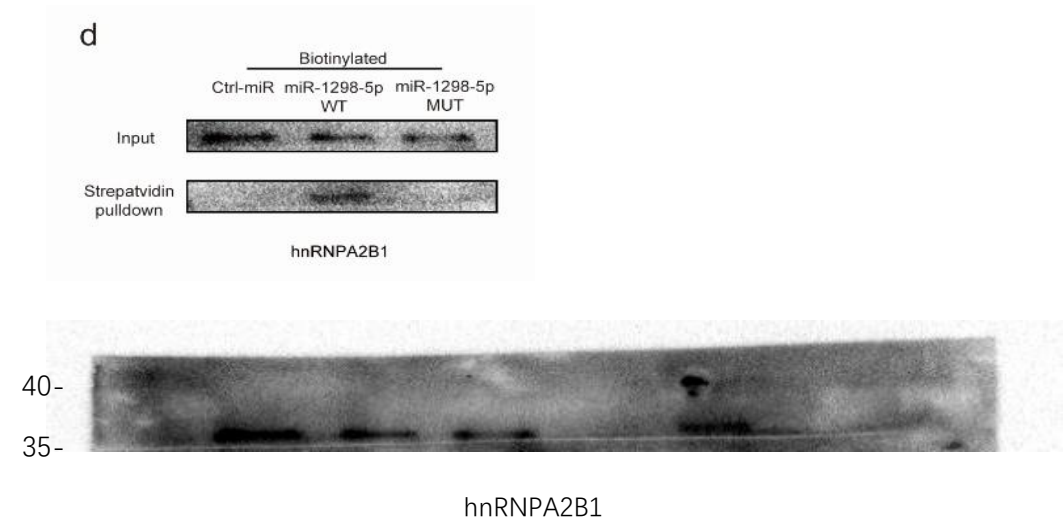

Figure5h

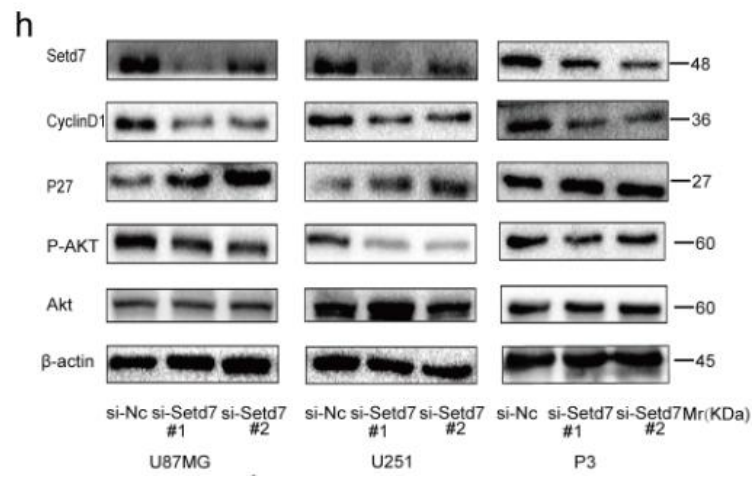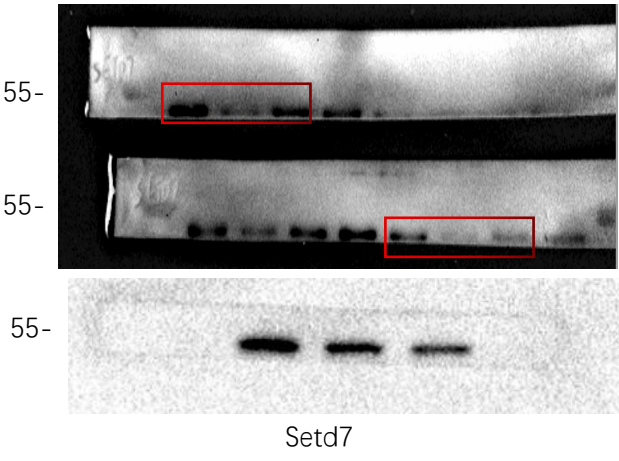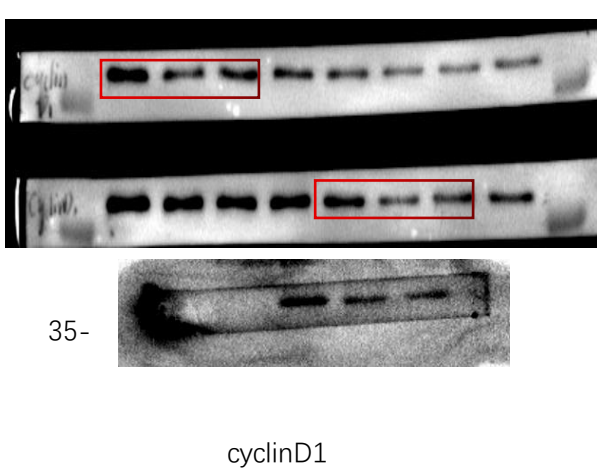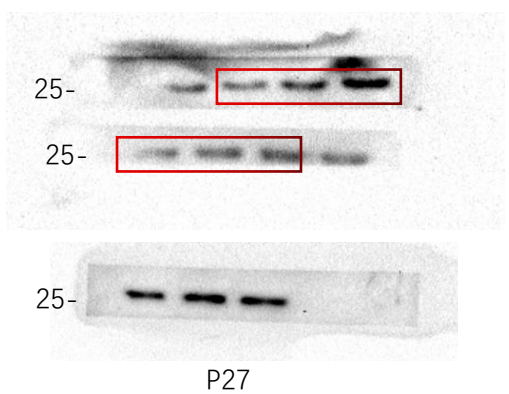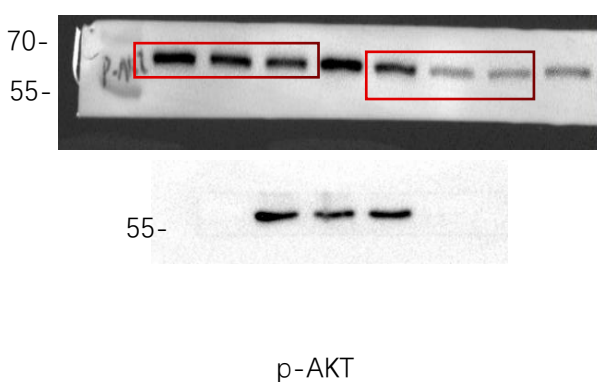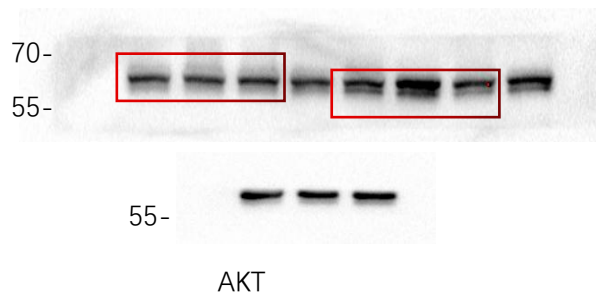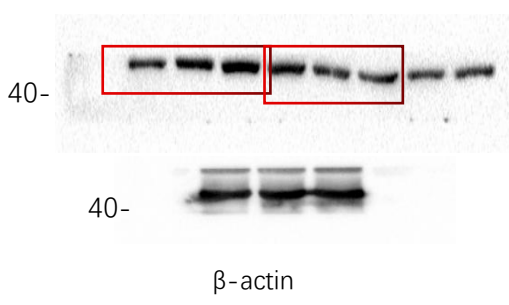

S

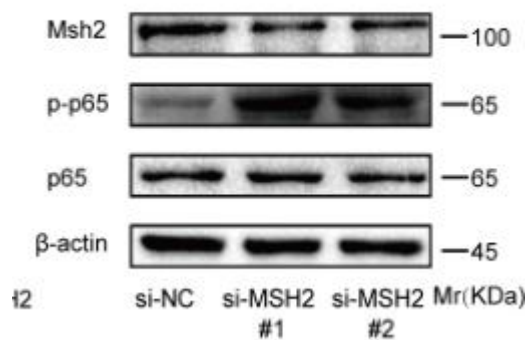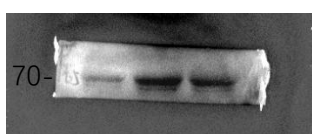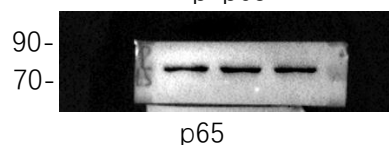

t

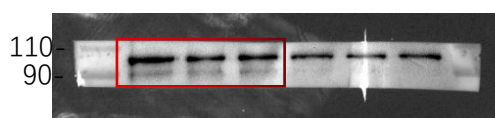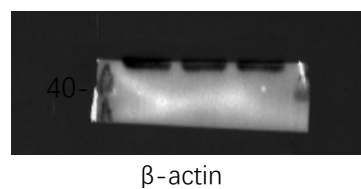

Figure5s

Figure7c

C

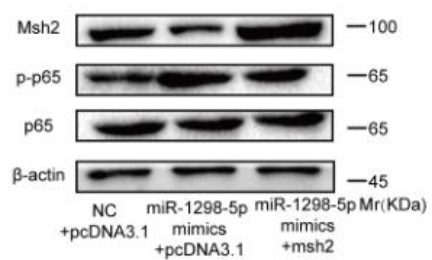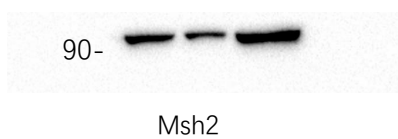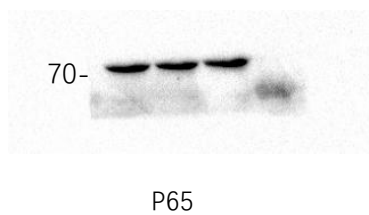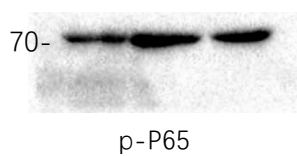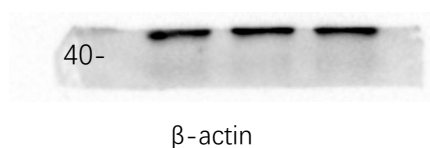

Figure6a

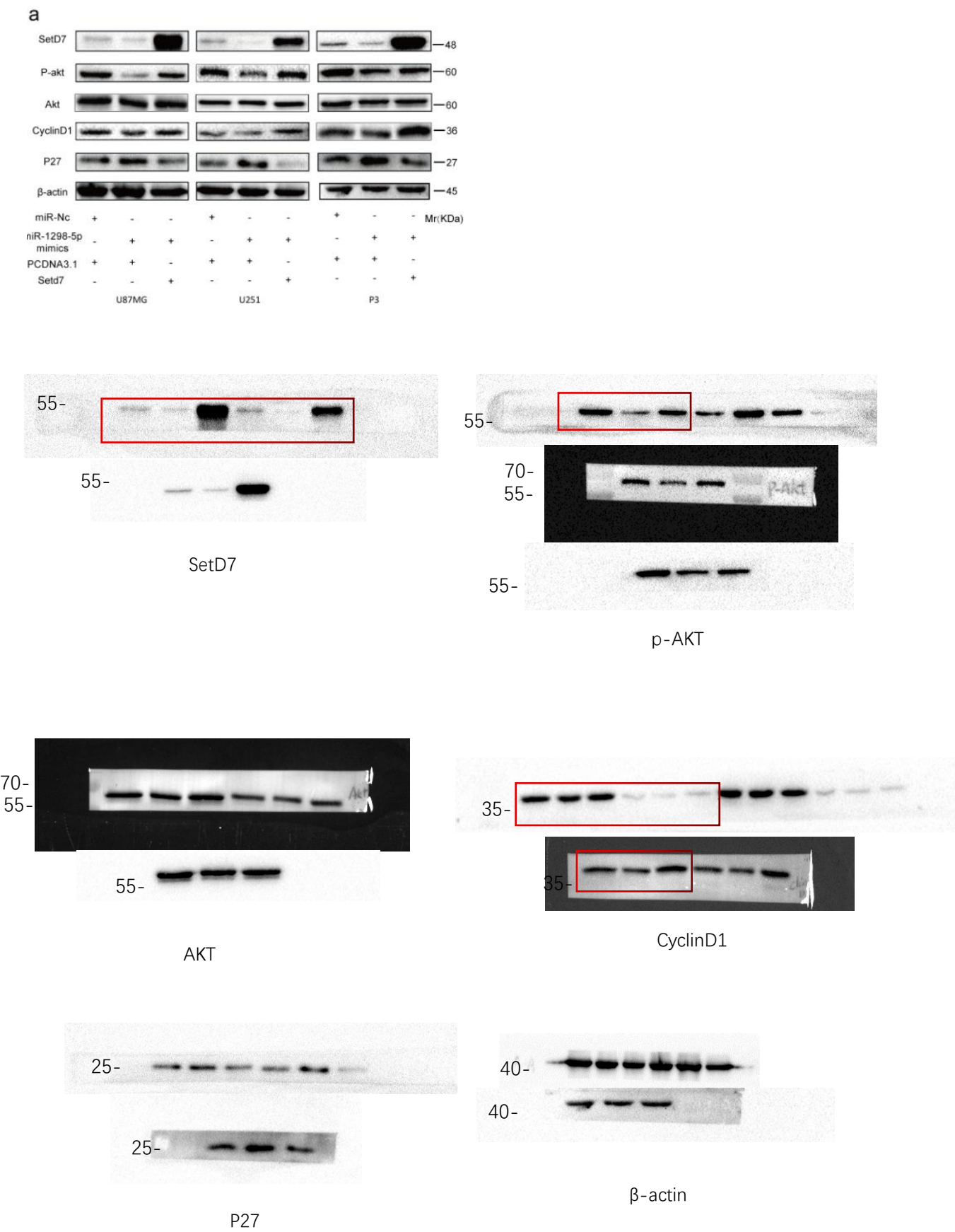

FigureS7j, n

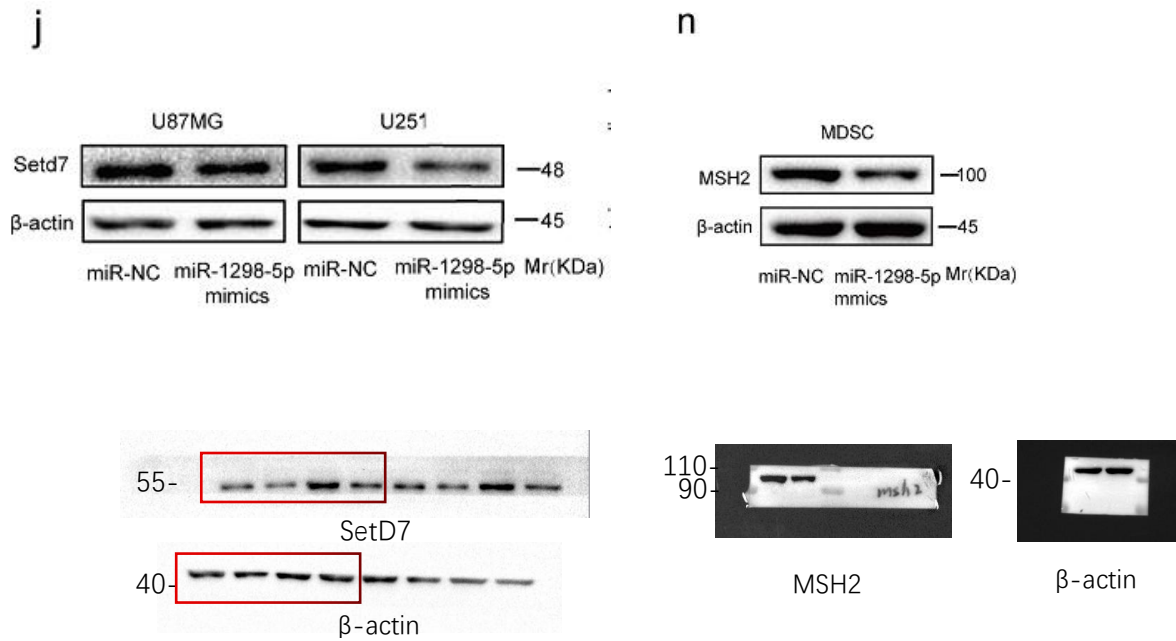

FigureS8b, f

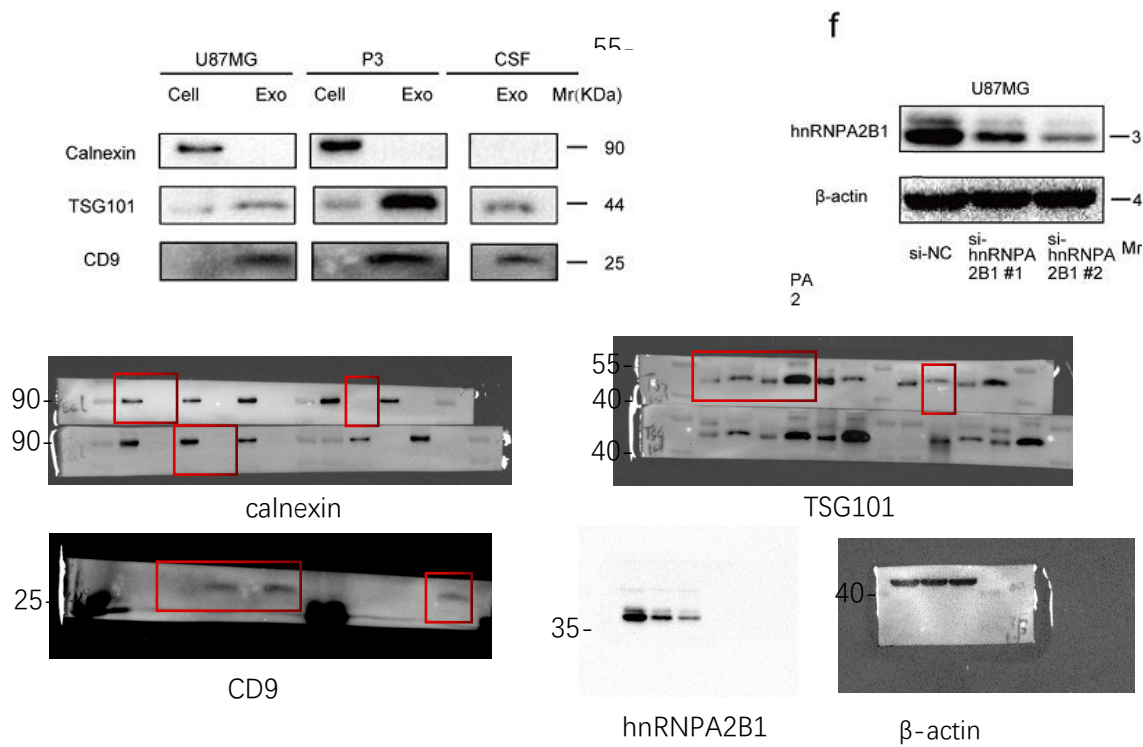

Supplement: Supplementary file 9 — uncropped westernblots [file 41419_2022_4872_MOESM9_ESM.pdf]
